# Endometrial receptivity in women of advanced age: an underrated factor in infertility

**DOI:** 10.1093/humupd/dmad019

**Published:** 2023-07-19

**Authors:** Amruta D S Pathare, Marina Loid, Merli Saare, Sebastian Brusell Gidlöf, Masoud Zamani Esteki, Ganesh Acharya, Maire Peters, Andres Salumets

**Affiliations:** Department of Obstetrics and Gynaecology, Institute of Clinical Medicine, University of Tartu, Tartu, Estonia; Department of Obstetrics and Gynaecology, Institute of Clinical Medicine, University of Tartu, Tartu, Estonia; Competence Centre on Health Technologies, Tartu, Estonia; Department of Obstetrics and Gynaecology, Institute of Clinical Medicine, University of Tartu, Tartu, Estonia; Competence Centre on Health Technologies, Tartu, Estonia; Division of Obstetrics and Gynaecology, Department of Clinical Science, Intervention and Technology (CLINTEC), Karolinska Institute and Karolinska University Hospital, Stockholm, Sweden; Department of Gynecology and Reproductive Medicine, Karolinska University Hospital, Stockholm, Sweden; Division of Obstetrics and Gynaecology, Department of Clinical Science, Intervention and Technology (CLINTEC), Karolinska Institute and Karolinska University Hospital, Stockholm, Sweden; Department of Clinical Genetics, Maastricht University Medical Centre+, Maastricht, The Netherlands; Department of Genetics and Cell Biology, GROW School for Oncology and Reproduction, Maastricht University, Maastricht, The Netherlands; Division of Obstetrics and Gynaecology, Department of Clinical Science, Intervention and Technology (CLINTEC), Karolinska Institute and Karolinska University Hospital, Stockholm, Sweden; Department of Clinical Medicine, Women’s Health and Perinatology Research Group, UiT The Arctic University of Norway, Tromsø, Norway; Department of Obstetrics and Gynaecology, Institute of Clinical Medicine, University of Tartu, Tartu, Estonia; Competence Centre on Health Technologies, Tartu, Estonia; Department of Obstetrics and Gynaecology, Institute of Clinical Medicine, University of Tartu, Tartu, Estonia; Competence Centre on Health Technologies, Tartu, Estonia; Division of Obstetrics and Gynaecology, Department of Clinical Science, Intervention and Technology (CLINTEC), Karolinska Institute and Karolinska University Hospital, Stockholm, Sweden

**Keywords:** endometrial aging, endometrial receptivity, cellular senescence, decidualization, oocyte donation, epigenetics

## Abstract

**BACKGROUND:**

Modern lifestyle has led to an increase in the age at conception. Advanced age is one of the critical risk factors for female-related infertility. It is well known that maternal age positively correlates with the deterioration of oocyte quality and chromosomal abnormalities in oocytes and embryos. The effect of age on endometrial function may be an equally important factor influencing implantation rate, pregnancy rate, and overall female fertility. However, there are only a few published studies on this topic, suggesting that this area has been under-explored. Improving our knowledge of endometrial aging from the biological (cellular, molecular, histological) and clinical perspectives would broaden our understanding of the risks of age-related female infertility.

**OBJECTIVE AND RATIONALE:**

The objective of this narrative review is to critically evaluate the existing literature on endometrial aging with a focus on synthesizing the evidence for the impact of endometrial aging on conception and pregnancy success. This would provide insights into existing gaps in the clinical application of research findings and promote the development of treatment options in this field.

**SEARCH METHODS:**

The review was prepared using PubMed (Medline) until February 2023 with the keywords such as ‘endometrial aging’, ‘receptivity’, ‘decidualization’, ‘hormone’, ‘senescence’, ‘cellular’, ‘molecular’, ‘methylation’, ‘biological age’, ‘epigenetic’, ‘oocyte recipient’, ‘oocyte donation’, ‘embryo transfer’, and ‘pregnancy rate’. Articles in a language other than English were excluded.

**OUTCOMES:**

In the aging endometrium, alterations occur at the molecular, cellular, and histological levels suggesting that aging has a negative effect on endometrial biology and may impair endometrial receptivity. Additionally, advanced age influences cellular senescence, which plays an important role during the initial phase of implantation and is a major obstacle in the development of suitable senolytic agents for endometrial aging. Aging is also accountable for chronic conditions associated with inflammaging, which eventually can lead to increased pro-inflammation and tissue fibrosis. Furthermore, advanced age influences epigenetic regulation in the endometrium, thus altering the relation between its epigenetic and chronological age. The studies in oocyte donation cycles to determine the effect of age on endometrial receptivity with respect to the rates of implantation, clinical pregnancy, miscarriage, and live birth have revealed contradictory inferences indicating the need for future research on the mechanisms and corresponding causal effects of women’s age on endometrial receptivity.

**WIDER IMPLICATIONS:**

Increasing age can be accountable for female infertility and IVF failures. Based on the complied observations and synthesized conclusions in this review, advanced age has been shown to have a negative impact on endometrial functioning. This information can provide recommendations for future research focusing on molecular mechanisms of age-related cellular senescence, cellular composition, and transcriptomic changes in relation to endometrial aging. Additionally, further prospective research is needed to explore newly emerging therapeutic options, such as the senolytic agents that can target endometrial aging without affecting decidualization. Moreover, clinical trial protocols, focusing on oocyte donation cycles, would be beneficial in understanding the direct clinical implications of endometrial aging on pregnancy outcomes.

## Graphical Abstract

**Figure:**
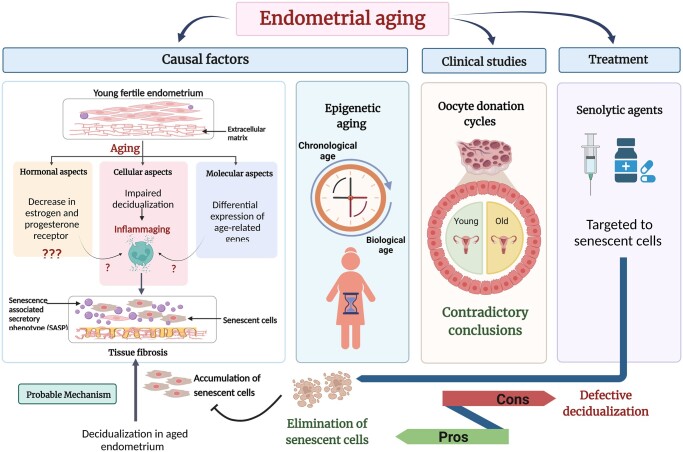
**Advanced age may negatively affect the endometrial functioning, therefore further research is needed to understand the exact mechanisms involved and impact on receptivity and pregnancy outcomes** Created with BioRender.com (https://biorender.com/).

## Introduction

Today, the average maternal age at childbirth has risen owing to the wider use of contraception and changes in various socio-economic and lifestyle-related factors, such as the educational and professional growth of women, housing and economic uncertainty, unmarried cohabitation before the birth of a first child, improved gender equity, etc. (Mills *et al.*, 2011). Additionally, the advent of novel and more effective ART has encouraged women to postpone childbearing. Statistically, the average childbearing age has increased by ∼1 year every decade since 1970 across the Organisation for Economic Co-operation and Development countries, with the average years of postponement ranging from 1.5 to 5 years (Mills *et al.*, 2011). During the preceding decade, advanced maternal age was considered as being over 35 years of age; however, based on the literature, the threshold has been lifted to 40 years, beyond which a steep decrease in fertility occurs even in IVF cycles (Shapiro *et al.*, 2016; Bouzaglou *et al.*, 2020). Different definitions have been used in the literature to describe advanced maternal age; however, because of the heterogeneity of study designs and the failure to control for potential confounders, the exact age indicating a decline in female fertility is still elusive. Nevertheless, age-related female infertility was reported to be clinically relevant from the age of 35–40 years (Devesa-Peiro *et al.*, 2022). Traditionally, the depletion of female fecundity with advanced age has been attributed to its evident association with ovarian dysfunction with a decrease in ovarian reserve leading to poor quality oocytes, eventually resulting in non-implantable embryos and even chromosomal abnormalities (Cimadomo *et al.*, 2018). However, age-related decline in fertility is not always limited to ovarian aspects. The currently available ART-related advances, such as oocyte donation and the selection of competent embryos by screening for chromosomal abnormalities using preimplantation genetic testing, have overcome the ovary-related aspects caused by advanced age to some extent. However, other factors, most importantly the aging of the endometrium, also influence the implantation rate, clinical pregnancy rate, and live birth rate in women of advanced age. This has been predominantly investigated in IVF studies using oocyte donation cycles. However, the variability in study design, selection and characteristics of oocyte donors, the clinical and demographic background of recipients, as well as the effect of other confounding factors, have led to controversial inferences about the effectiveness of oocyte donation in advanced-age patients. Moreover, several reports on endometrial aging have been published in discrete areas using animal models and human studies to determine the effect of uterine aging on the endometrial ultrasound parameters such as thickness, histological and cellular senescence in endometrial tissue, levels of steroid hormones, distribution of their receptors in endometrial tissue, and the molecular determinants of endometrial decidualization and receptivity. A recently published review postulates the significance of age-related epigenetic alterations and future implementation of the epigenetic clock to predict the biological age of endometrium by virtue of related disorders and infertility (Deryabin and Borodkina, 2023). Another recently published review article summarizes the overall functional and structural alterations, with probable mechanisms, involved in uterine aging (Wu *et al.*, 2023). However, the current review is intended to compile the available literature on endometrial aging with the prime focus on synthesizing evidence regarding the impact of aging on the success of conception and establishment of a pregnancy. This aspect of endometrial aging has been comprehensively covered in our review, supported by different types of clinical studies with oocyte donation cycles. Thus, we emphasize the clinical significance of endometrial aging, which contribute to the uniqueness of our review. Besides, the review describes those hormonal, cellular, molecular, and epigenetic factors causing an age-related impact on endometrial receptivity. Lastly, the challenges in the implementation of anti-aging therapies, such as senolytic agents, to reverse age-associated endometrial impairments are discussed.

## Methods

### Search strategy

A comprehensive narrative literature review was conducted based on a search of articles in PubMed (Medline) until February 2023. An overview of the cellular, hormonal, molecular, and epigenetic aspects of healthy endometrium and the associated alterations as an effect of endometrial aging was acquired from the available research studies using animal models as well as human patients and samples. Clinical studies were included to understand the impact of aging on endometrial receptivity among oocyte donation cycles. These studies were segregated into three groups based on the study design and selection criteria for cases and controls: oocytes donated by healthy oocyte donors which were transferred to recipients of younger or advanced age; a single pool of oocytes donated by a young woman and distributed among the recipients with young and advanced age; and infertile patients undergoing IVF who had shared their oocytes with other recipients of advanced age, i.e. standard IVF with own oocytes versus IVF with donated oocytes. Further, knowledge regarding senolytic agents and the associated challenges involved in the implementation of these therapies in alleviating age-related endometrial dysfunction was highlighted using available scientific reports, primarily on animal model studies. The keyword endometrial/uterine aging was searched alone and in combination with ‘receptivity’ and other terms: ‘decidualization’, ‘hormone’, ‘senescence’, ‘cellular’, ‘molecular’, ‘methylation’, ‘biological age’, ‘epigenetic’, ‘oocyte recipient’, ‘oocyte donation’, ‘embryo transfer’, and ‘pregnancy rate’. Duplicate articles were eliminated from consideration.

### Data screening and eligibility criteria

From the primary search, retrospective, prospective, and cohort clinical studies published in English only were included. Publications with animal studies and human cases were included wherein the age groups corresponding to reproductive age were compared to indicate their impact on endometrial receptivity. The age groups outside the reproductive years were not considered in this review and were excluded using the keywords ‘post-menopause’, ‘menarche’ and ‘age over 50’, ‘age over 60’. Additionally, since the current review focuses on the effect of aging on pregnancy outcomes and is restricted to conception as the outcome measure, those articles having pregnancy-related complications as the primary outcome measures were also excluded. To ensure an unbiased evaluation of the impact of advanced age on endometrial receptivity, articles concerning cancer, endometriosis, and other endometrial disorders were excluded. Relevant citations and cross-references were also screened and examined.

### Data extraction and study selection

The screening of articles was performed independently by two authors by evaluating the titles and abstracts. Any discrepancies were resolved by discussion among other authors. Irrelevant articles were eliminated using the Newcastle-Ottawa Quality Assessment Scale (Lo *et al.*, 2014). When an article was considered suitable for inclusion, the full-text version was referred to. Animal model studies complying with Animal Research: Reporting of In Vivo Experiments (ARRIVE) guidelines were considered for this review (Kilkenny *et al.*, 2010). The quality of clinical studies was evaluated using the Appraisal Tool for Cross-Sectional Studies (AXIS) guidelines (Downes *et al.*, 2016). For each publication, the data were manually extracted, including study type, publication date, aspect of aging, the origin of samples used, sample size, age groups, comparison groups, outcome measures, and the effect(s) of aging on endometrial biology. In the case of oocyte donation studies, the articles in each group were segregated as per the negative or no impact of endometrial aging based on clinical pregnancy, implantation, and delivery rates. Owing to the absence of sufficient data for a meta-analysis, the studies are summarized here in the text and in tables.

## Results

**Figure 1.**
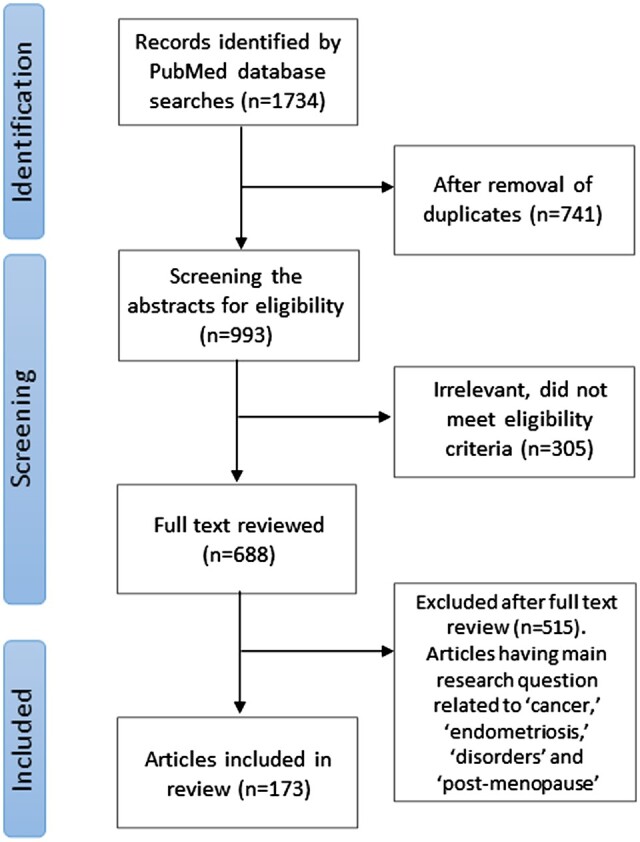
**Method flow chart illustrating the identification and screening of literature for this narrative review of aging and the endometrium**.

A total of 1734 articles were identified after the primary search, of which 993 were retained after eliminating duplicates. On screening the abstracts for eligibility, 688 articles were evaluated. Further, 515 publications corresponding to the main objectives of ‘cancer’, ‘endometriosis’, ‘disorders’, and ‘menopause’ were removed, and a total of 173 relevant manuscripts were selected and studied for the current review, of which 14 were animal model studies and 24 were clinical studies of oocyte donations. Randomized controlled trials were not available on this subject. The selection flow chart is shown in Fig. 1.

## Functioning of healthy endometrium during the reproductive years

The endometrium is a very complex tissue consisting of different cell types, e.g. stromal, epithelial, vascular, and immune cells. The endometrium is functionally divided into two layers—‘functionalis’ and ‘basalis’. Functionalis is the superficial layer of endometrium that undergoes cyclic changes as per the different phases of the menstrual cycle, which are predominantly regulated by the ovarian steroid hormones, estradiol (E2), and progesterone (P4). Histologically, this layer consists of a simple columnar endometrial surface epithelium called the luminal epithelium. The glandular epithelium forms several tubular glands and is surrounded by thick vascular stroma comprising fibroblast-like stromal cells and spiral arteries, which recruit the population of fluctuating innate immune cells. Endometrium functionalis sheds off during menstruation, regenerates during the proliferative phase, and matures during the secretory phase in order to achieve receptive potential for the implanting embryo (Jiménez-Ayala and Jiménez-Ayala Portillo, 2008). In the absence of fertilization, in response to the withdrawal of P4, it undergoes the successive cycle of menstruation.

Endometrium basalis is a thin basal layer that adjoins the myometrium and is located beneath the functionalis layer. In contrast to functionalis, it consists of permanent stroma and endometrial glands, which do not tend to undergo regular cyclic changes (Deryabin *et al.*, 2020). The stroma in the basal layer is denser with an increased nucleocytoplasmic ratio and thicker-walled arteries than the endometrium functionalis. In humans, the basalis layer exhibits the presence of progenitor stem cells, which can differentiate into stromal and epithelial cells (Critchley *et al.*, 2020). Thus, it performs a vital role in maintaining endometrial integrity across every menstrual cycle (Jiménez-Ayala and Jiménez-Ayala Portillo, 2008).

In the normal menstrual cycle of reproductive-aged women, the proliferative phase is characterized by the proliferation of endometrial cells synchronized with follicular growth in response to increasing levels of E2. The action of E2 is mediated by genomic and non-genomic E2 signalling pathways via their corresponding nuclear E2 receptors (ERs, ERα, and ERβ) and membrane-associated ERs (ERα36 and G protein-coupled E2 receptor (GPER)), respectively (Pagano *et al.*, 2020; Yu *et al.*, 2022). During this phase, the luminal epithelium is thinner and consists of tall columnar cells. Endometrial glands are straight and narrow and become torturous towards the end of the proliferative phase. Glands exhibit signs of mitosis and pseudo-stratification of glandular epithelium. Stroma is compact and dense, having small stromal cells with a comparatively large nucleus and scanty cytoplasm undergoing mitosis (Noyes *et al.*, 1950). After ovulation, the E2 is opposed by elevated P4 levels produced by the corpus luteum, which promotes the differentiation of endometrial cells forming thick, mature, and blood vessel-rich tissue defining the secretory phase of the menstrual cycle. Secretory endometrium is characterized by larger, more tortuous glands lined by low columnar cells, an absence of mitosis, and pseudo-stratification (Noyes *et al.*, 1950). The nuclei of glandular epithelium are observed to be shifted at the base of cells with the presence of subnuclear cytoplasmic glycogen vacuoles contributing to secretion into the lumen of glands, causing enlargement. The secretory phase is presented with stromal oedema underlying the luminal epithelium with the diffused appearance of the walls of spiral arteries. During the mid-secretory phase, under the influence of ovarian P4 and cyclic AMP, the endometrial stromal cells undergo a cellular differentiation and transformation into the decidual stromal cells accompanied by changes of the cellular population in the endometrial tissue; the process is characterized as decidualization (Okada *et al.*, 2018). Decidualization is associated with the recruitment of leukocytes and immune cells at the decidua, which collectively secretes cytokines, chemokines, and several growth factors, making the endometrium receptive to an implanting embryo (Ng *et al.*, 2020). Decidualization is responsible for opening the window of implantation (WOI), which is the self-limiting period that typically occurs 6–7 days following the LH surge and continues for approximately 4 days, within which the event of embryo implantation may take place (Achache and Revel, 2006; Koot *et al.*, 2012). The transformation from early-secretory to mid-secretory endometrium is regulated at the molecular level resulting in alterations in gene expression (Altmäe *et al.*, 2017). Further, with the diminishing level of P4, the endometrium shows degeneration of glandular cells and displacement of secretory glands and stromal aggregates causing tissue haemorrhage, and the superficial functional layer separates from the basal layer, causing menstruation. Thus, the endometrium exhibits cellular and histological changes in response to fluctuations in the levels of hormones across the menstrual cycle (Noyes *et al.*, 1950; Gellersen and Brosens, 2014; Garcia-Alonso *et al.*, 2021).

## Effect of endometrial aging: hormonal, cellular, and molecular aspects

### Effect on endometrial aging: hormonal aspects

During the normal menstrual cycle, two distinct peaks of serum E2 levels are observed. The first peak occurs during the proliferative phase and is associated with the increased expression of endometrial E2 receptors ERα, ERβ, and GPER inducing mitogenic activity (Giudice, 2006; Yu *et al.*, 2022). The progesterone dominated secretory phase is accompanied by lower levels of estradiol and ERα than the proliferative phase (Dockery and Rogers, 1989; Critchley *et al.*, 2020; Yu *et al.*, 2022). During the mid-secretory phase, the second peak of E2 coincides with the WOI and increasing levels of P4. The action of P4 stimulates the decidualization process that enables the receptivity status of the endometrium (Yu *et al.*, 2022). In the case of successful embryo implantation, the pregnancy is maintained in response to the higher levels of E2 and P4. In the absence of implantation, decreased levels of E2 and P4 lead to the initiation of the menstrual phase (Ruiz-Alonso *et al.*, 2012; Critchley *et al.*, 2020).

The serum concentration of E2 across the ovulatory menstrual cycles is variable among women of advanced age. According to the studies performed, the average serum E2 concentrations decreased with age from 42–45 to 52–55 years, with a steep decline towards higher ages (Burger *et al.*, 1999; Randolph *et al.*, 2004). However, another cross-sectional study showed similar follicular E2 levels among women in early and mid-reproductive age (21–45 years) as well as for the age group of >45 years (Hale *et al.*, 2007). Conversely, a separate study (Burger *et al.*, 2008) during ovulatory cycles revealed that the serum E2 levels in the follicular phase were elevated in older women (>45 years) compared to younger women (21–35 years). This age-related unexpected increase in E2 levels in the follicular phase were explained by the hypothesis that the feedback mechanism operating via inhibin B secretion is affected earlier than E2 secretion. As follicle numbers decrease with age, it first affects the inhibin B secretion instead of E2, leading to declined levels of inhibin B, which is associated with the consequent increase in FSH levels. This, eventually, can result in elevating E2 production by granulosa cells (Burger *et al.*, 2008). Thus, the evidence implies that advanced age affects the hypothalamic pituitary–ovarian axis, with fluctuating levels of E2 in the follicular phase of ovulatory cycles in the late-reproductive stage compared to younger women. The close correlation between E2 levels and endometrial proliferation suggests that abnormal E2 concentrations in older women may have a detrimental effect on endometrial growth and thickness. Moreover, during the secretory phase, serum levels of E2 and P4 decrease progressively with age (MacNaughton *et al.*, 1992), which further can influence cellular differentiation during endometrial receptivity.

The age-dependent variation in E2 and P4 levels may have a considerable impact on the endometrium by altering the endometrial expression of their corresponding cellular receptors. Furthermore, the age-related endometrial downregulation of ER and P4 receptors (PR) has been demonstrated in the endometrial tissue. Additionally, the decreased capacity of the endometrium to take up these steroids (Larson *et al.*, 1972; Blaha and Leavitt, 1974; Saiduddin and Zassenhaus, 1979) was associated with increased collagen and fibrosis in endometrial tissue (Ohta, 1987; Nelson *et al.*, 2013). The mouse model study also exhibited impaired hormonal responsiveness in aged mice as around 50% of de-regulated genes in aged decidua were associated with ERα and/or PR. Additionally, expression of ER and PR was more variable in aged mice showing mosaic staining patterns in luminal epithelium versus homogenous expression in young mice (Woods *et al.*, 2017). In human endometrium, the expression of cytosolic ER has been reported to change with age, being highest in the 30–39 years age group compared to the 20–29 years and 40–55 years age groups. On the other hand, the nuclear ER and PR showed an age-dependent decrease in the endometrium of women aged 20–29, 30–39, 40–49, and above 50 years (Bergqvist and Ferno, 1993). Furthermore, the staining of epithelial cell nuclei positive for ERα was 100% on the day of oocyte retrieval among healthy oocyte donors aged <30 years, which significantly declined to 90% staining among women >30 years of age, showing an age-related decline in the expression of ERα (Klonos *et al.*, 2020). In contrast, another study showed similar expression of ER and PR in both age groups of women <30 years and >40 years, indicating that there was no effect of age on the endometrial function until menstruation is present (Noci *et al.*, 1995). Similarly, functionally agonadal women in the different age groups from 25 to 60 years showed a similar effect of hormone replacement therapy on histologic and ultrasonographic characteristics of the endometrium, as well as expression of ER and PR, regardless of their age (Sauer *et al.*, 1993). Thus, more detailed age-dependent studies on the levels and distributions of steroid hormone receptors are needed to determine their causal effect on endometrial aging. Nevertheless, empirical evidence suggests abnormal levels of ovarian hormones and their receptors in older women, indicating the direct influence on endometrial proliferation, receptivity, and overall functioning.

#### Endometrial thickness and aging

E2 and P4 are responsible for achieving the desired endometrial thickness, which is a pre-requisite for the implanting embryo. Endometrial morphology can be assessed using ultrasonographic techniques to measure the thickness and study the echogenic pattern of the endometrium, both of which vary during each phase of the natural menstrual cycle in healthy fertile women (Fitzgerald *et al.*, 1994). Although there is no consensus about the minimum endometrial thickness that is favourable for the implantation of the embryo, clinical pregnancy has been achieved in women with endometrial thickness as low as 4 mm and as high as 16 mm (Ugwu *et al.*, 2022). On the other hand, an endometrial thickness of 7 mm has been reported to be a pre-requisite for implantation (Tomic *et al.*, 2020; Ugwu *et al.*, 2022), followed by an increase in implantation rate when the endometrium increases from 8 to 11 mm, whereas when the endometrial thickness increases to >14 mm, it showed a dramatic decrease in implantation rate (Eftekhar *et al.*, 2019; Ugwu *et al.*, 2022). The assessment of endometrial thickness in different age groups of healthy women revealed that the endometrium was significantly thicker during the mid-secretory phase compared to ovulation day in all the age groups. Further, it was observed that in women aged 32–36 years and 37–45 years, the maximum thickness reached during the secretory phase was 15.3 and 15.9 mm, respectively, which was significantly higher than in younger age groups of 21–25 and 26–31 years showing a maximum thickness of 12.1 and 13.4 mm, respectively (Fitzgerald *et al.*, 1994). However, the extrapolation of this age-related difference in endometrial thickness and its effect on pregnancy rate has not been established. In IVF cycles, studies have reported a positive correlation of endometrial thickness with pregnancy rate (Richter *et al.*, 2007; Yang *et al.*, 2018) whereas other studies did not find any significant association between these parameters (Žáčková *et al.*, 2009; Gingold *et al.*, 2015).

Some studies suggest that endometrial patterns, such as triple-line configuration, patterns of a hyperechoic endometrium, and the presence of a central echogenic line detected by ultrasound as well as by the evaluation of endometrial blood flow, have more impact on the implantation potential as compared to endometrial thickness (Gonen and Casper, 1990; Järvelä *et al.*, 2005; Gingold *et al.*, 2015; Navinchandra *et al.*, 2016). Studies have also reported the combined approach of endometrial pattern together with thickness, and a scoring system for the prediction of pregnancy success during IVF (Baruffi *et al.*, 2002; Chen *et al.*, 2010; Gingold *et al.*, 2015; Khan *et al.*, 2016; Navinchandra *et al.*, 2016; Ugwu *et al.*, 2022; Zhang *et al.*, 2022). An IVF study reported that the triple-line endometrial pattern observed on the day of the administration of hCG was associated with an improved pregnancy rate compared to the homogenous, hyperechogenic, or intermediate endometrial pattern (Gingold *et al.*, 2015). However, as for endometrial thickness, there are conflicting conclusions even on the correlation of endometrial pattern with pregnancy rate (Gonen and Casper, 1990; Check *et al.*, 1993b; Järvelä *et al.*, 2005; Potlog-Nahari *et al.*, 2005; Rashidi *et al.*, 2005; Mercé *et al.*, 2008; Ugwu *et al.*, 2022), without the information on age-related shift. The endometrial thickness and pattern can be used as prognostic indicators for endometrial receptivity; however, these features have not been well accepted as sole predictive markers for successful implantation (Navinchandra *et al.*, 2016). Additionally, detailed information regarding age-related alterations in endometrial morphology and thickness is not yet available. Since these parameters have direct clinical implications, further precise research studies and randomized controlled clinical trials evaluating the impact of age on endometrial thickness and echogenic pattern, and their correlation with pregnancy outcome, would prove to be constructive.

### Effect on endometrial aging: cellular aspects

#### Tissue aging and cellular senescence

**Figure 2.**
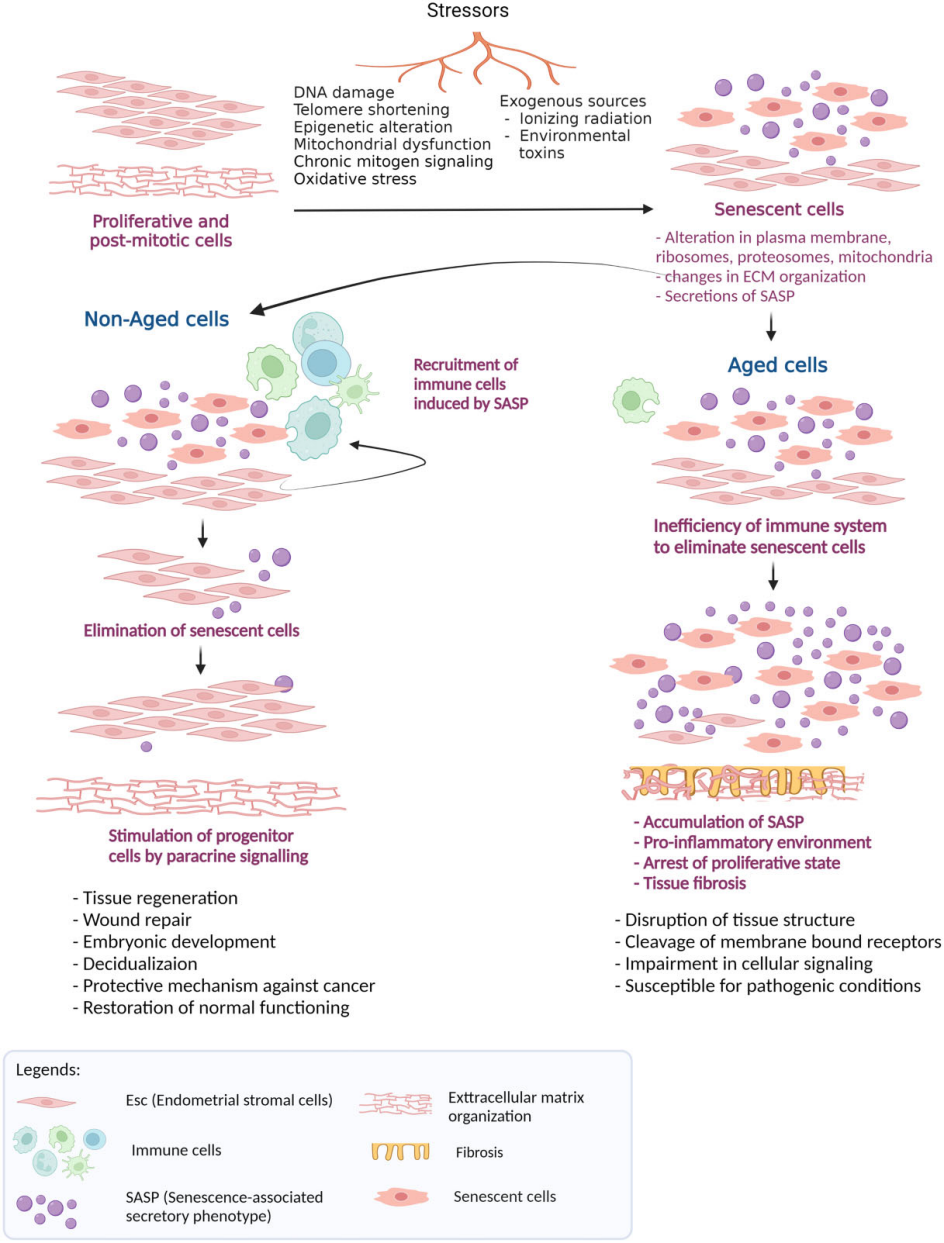
**Cellular senescence in normal and aged cells.** The phenomenon of cellular senescence occurs in different types of human tissues, including endometrium. It involves the transformation of both proliferative and post-mitotic cells into senescent cells in response to various types of stressors. These stimuli can be telomere shortening, epigenetic changes, DNA damage, oxidative stress, chronic mitogen signalling, and mitochondrial dysfunction over a period, which leads to cell proliferation arrest. Additionally, exogenous sources, such as ionizing radiation, ultraviolet light, and environmental toxins, can contribute as stressors resulting in cellular senescence. In normal conditions, the fully senescent cells are targeted by immune cells to be eliminated from the immune system, whereas during circumstances of aging the immune system is unable to clear senescent cells, which leads to the accumulation of senescence-associated secretory phenotype (SASP) and a persistent pro-inflammatory environment. Created with BioRender.com (https://biorender.com/).

Aging is a complex and multifactorial biological process that promotes time-dependent deterioration of tissue function across multiple organ systems. Aging is a result of cumulative changes caused by several stimuli, such as telomere shortening, epigenetic changes, DNA damage, oxidative stress, chronic mitogen signalling, and mitochondrial dysfunction over a period, which leads to cell proliferation arrest and a decline in cellular function (McHugh and Gil, 2018; Deryabin *et al.*, 2020; Di Micco *et al.*, 2021). Additionally, exogenous sources, including ionizing radiation and environmental toxins, can contribute as stressors resulting in cellular senescence (Fig. 2) (Todhunter *et al.*, 2018; Kumari and Jat, 2021; Yousefzadeh *et al.*, 2021). At the level of a single cell, aging is closely associated with the underlying biological process called cellular senescence. Cellular senescence is the irreversible cell cycle arrest of not only proliferative cells but also post-mitotic cells (Sapieha and Mallette, 2018; Anderson *et al.*, 2019; Maduro *et al.*, 2021). Apart from aging, cellular senescence can also be caused by the response to damage induced by stressors, even in non-aged cells (Todhunter *et al.*, 2018; Deryabin *et al.*, 2020). However, under the influence of an appropriate microenvironment, naturally occurring senescence in healthy cells attracts the immune cells to eliminate them to protect against possible tumour development (Deryabin *et al.*, 2020). Thus, not all senescent cells are aged cells, but cellular senescence is the main hallmark of the aging process.

Aging-associated cellular senescence modulates the cell mechanics and organization of the extracellular matrix (ECM) (Fig. 2). It alters the functioning of intracellular organelles, including the plasma membrane, ribosomes, proteosomes, mitochondria, and lysosomes, changes the morphology of nuclear structures, and dysregulates gene expression as well as epigenetic modifications of DNA and histones (Borodkina *et al.*, 2018; Ogrodnik *et al.*, 2019). Additionally, as a response to DNA damage, the senescent cells secrete several pro-inflammatory cytokines, chemokines, growth factors, and matrix metalloproteinases, which are collectedly termed the ‘senescence-associated secretory phenotype (SASP)’ (Borodkina *et al.*, 2018; Deryabin *et al.*, 2020; Maduro *et al.*, 2021). In a normal healthy scenario, controlled secretion of SASP is reported to be beneficial and allows the clearance of senescent cells by elicitation of the immune response and replacing them with adjacent progenitor cells via paracrine signalling to achieve tissue regeneration (Deryabin *et al.*, 2020; Deryabin and Borodkina, 2022). However, the accumulation of senescent cells in the aged tissues of humans, primates, and rodents has been demonstrated (Herbig *et al.*, 2006; Wang *et al.*, 2009; van Deursen, 2014), characterizing it as chronic senescence. A predominant drawback of this chronic aging process is the inefficiency of the body’s immune system to eliminate senescent cells, for various reasons: an increased rate of senescence with age; age-associated immunodeficiency in the innate and adaptive immune systems, reducing the potential for senescent-cell clearance; and dysfunction in haematopoietic stem cells with age, thus compromising the immune system (Fig. 2) (van Deursen, 2014). In this scenario, the senescent, aged cells continue to be increasingly dependent on the cell cycle checkpoints leading to the arrest of the proliferation state for a longer period, which is associated with the accumulation of SASP secretion (van Deursen, 2014; Deryabin *et al.*, 2020). The accumulated population of senescent cells and SASP results in disruption of the tissue structure, cleavage of membrane-bound receptors, impairment in cellular signalling, and dysfunction of the ECM. Elevated levels of pro-inflammatory cytokines, such as IL-6 and IL-8, stimulate chronic inflammation and the infiltration of macrophages and lymphocytes, and induces tissue fibrosis (Coppé *et al.*, 2008; Campisi, 2013; van Deursen, 2014). Thus, aging exhibits tissue-specific changes, which ultimately influence its functioning.

#### Association between decidualization and cellular senescence

**Figure 3.**
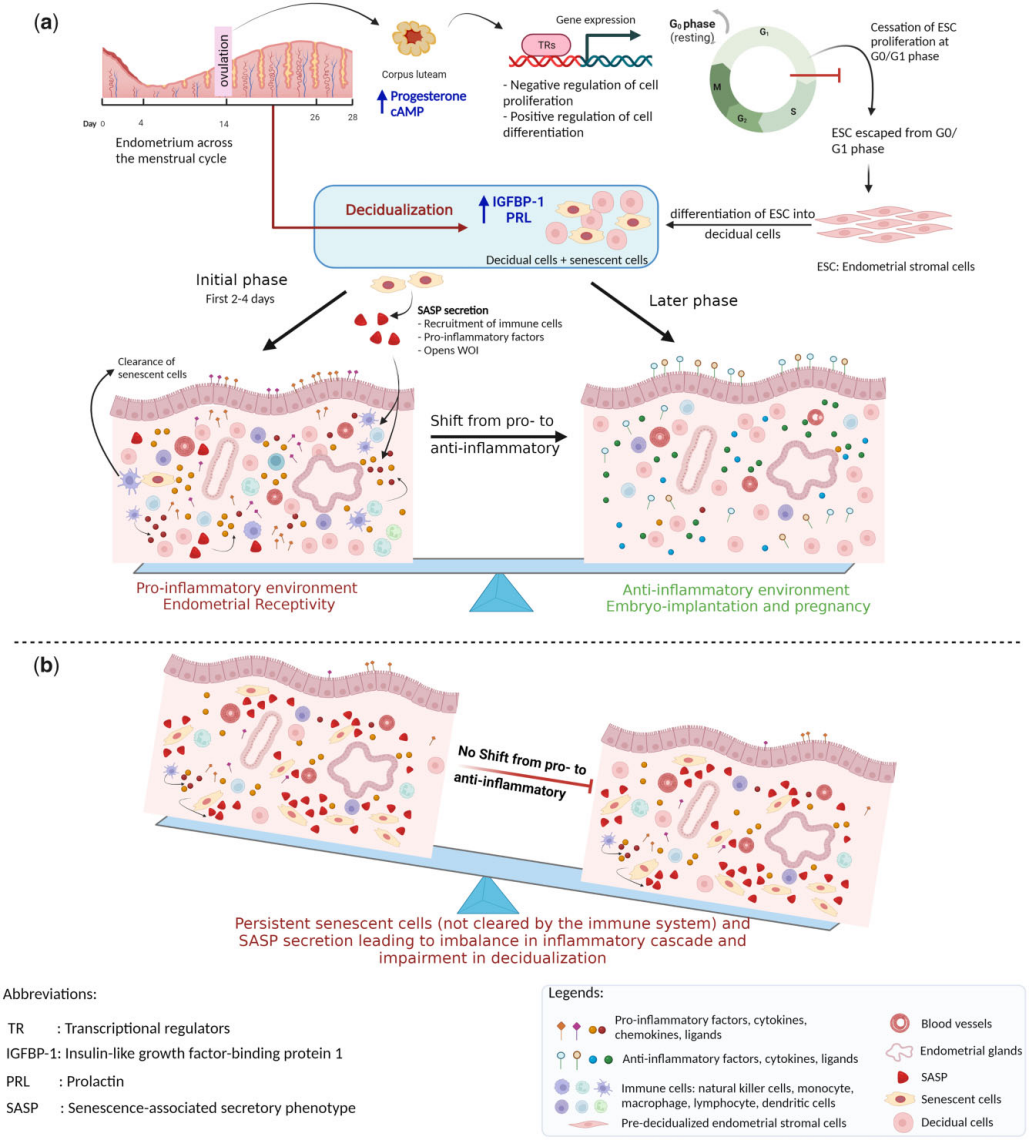
**Relation between decidualization and cellular senescence in normal conditions and during the aging process.** (**a**) Normal physiological conditions: in the mid-secretory phase, under the influence of P4 and cAMP, the endometrial stromal cells escape from the cell cycle at the G0/G1 phase and undergo cell differentiation pathways to transform into decidual cells, in the process called decidualization. In the initial phase during decidualization, the subpopulation of senescent cells is present along with the mature decidual cells. Senescence-associated secretory phenotype (SASP) manifested by the endometrial cells is essential for developing an adequate pro-inflammatory response required for the secretion of cytokines, chemokines, and cell adhesion ligands that are pre-requisites for embryo implantation. In the later phase, mature senescent cells are targeted by the immune cells, decreasing the levels of SASP and shifting the endometrial milieu to the anti-inflammatory response that is required to maintain a semi-allogeneic embryo during the pregnancy. (**b**) During the aging process: in a chronic condition like aging, the immune cells cannot eliminate the mature senescent cells during decidualization. This results in the accumulation of pre-senescent, fully senescent cells and secreted SASP causing persistent unregulated pro-inflammation during mid-secretory endometrium. This fails to maintain the inflammatory balance leading to impaired decidualization and defective endometrial receptivity. Created with BioRender.com (https://biorender.com/).

Decidualization involves several biological functions including cell differentiation, immune and inflammatory responses, transcription regulation, negative regulation of cell proliferation, coagulation, complement cascade, ECM organization, vascularization, and tissue remodelling (Okada *et al.*, 2018). In humans, decidualization occurs in every menstrual cycle irrespective of the presence of an embryo (Teklenburg *et al.*, 2010), unlike other species, such as rodents, where the signalling of the blastocyst initiates decidualization (Das, 2010). Thus, in humans, decidualization is solely induced by the maternal system through various transcription factors that mediate negative regulation of endometrial stromal cell (ESC) proliferation by cessation of the cell cycle at the G0/G1 phase (Rytkönen *et al.*, 2019; Deryabin *et al.*, 2020). It induces the endometrial expression of insulin-like growth factor-binding protein-1 (IGFBP-1) and prolactin, which are decidualization markers, and thus stimulates the differentiation of ESCs into decidual cells (Okada *et al.*, 2018) (Fig. 3a). This transformation opens the WOI and is accompanied by the recruitment of monocytes and other immune cells from the circulation including uterine natural killer (uNK) cells (Mori *et al.*, 2016). Endometrial decidual cells and immune cells secrete an array of pro-inflammatory mediators, such as tumour necrosis factor α, IL2, IL12, prokineticin 1, and interferon γ (IFNG), as well as chemokines, and cytokines such as C-X-C motif chemokine ligand 12 (CXCL12), CXCL14, leukemia inhibitory factor, IL1β, IL11, and IL-6. Additionally, other important factors involved in embryo implantation, such as growth factors like epidermal growth factor (EGF), heparin-binding EGF, insulin-like growth factor 1 (IGF1), and morphogenesis proteins Indian hedgehog, Wnt family member 4, and bone morphogenetic protein 2 (BMP2) also play crucial roles (Achache and Revel, 2006; Ruiz-Alonso *et al.*, 2012). Moreover, the nuclear factor kB-dependent signalling pathways via ER are activated to promote cellular inflammation during the WOI (King *et al.*, 2010). Although the proinflammatory milieu in the endometrium during decidualization is essential, it is a time-dependent and well-regulated phase limited to the initiation of WOI. Later, the decidual process shifts from the expression of a pro-inflammatory to an anti-inflammatory profile, to secrete the anti-inflammatory hormone cortisol, other anti-inflammatory factors, and cytokines, such as IL-4, IL-5, and IL-10 (Deryabin *et al.*, 2020), which protect the semi-allogeneic embryo from the maternal immune system (Fig. 3a). An anti-inflammatory condition is thus maintained until delivery in case of a successful pregnancy; otherwise, in the absence of fertilization, a reducing level of P4 triggers the vasoconstriction of blood vessels and induces menstrual bleeding.

The induction of successful decidualization is associated with the presence of senescence markers in decidual ESCs (Deryabin *et al.*, 2020). Senescence-specific protein p16 was upregulated in a subpopulation of ESCs, which was characterized as senescent decidual cells because they expressed genes involved in the cellular senescence pathway (Brighton *et al.*, 2017). Furthermore, the study infers that an adequate degree of senescence is responsible for producing a sub-population of senescent decidual cells, which is a pre-requisite for the decidualization process. A recent study using single-cell RNA sequencing confirmed the presence of both mature decidual cells along with a senescent decidual subpopulation that occurred after normal ESC decidualization (Lucas *et al.*, 2020). Thus, the initial pro-inflammatory response during decidualization is the result of the presence of mature, as well as senescent, decidual cells. The intensity of this pro-inflammatory response depends on the degree of the pre-senescent population of ESCs (Deryabin *et al.*, 2020; Lucas *et al.*, 2020). Senescent decidual cells also secret IL15, which is one of the essential decidualization markers, that activates peripheral NK cells and transforms them into tissue-resident uNK cells. Transformed uNK cells are CD54+ and CD16-, without their cytotoxicity property, which are essential for vascularization and spiral artery remodelling during the implantation and placentation process (Jabrane-Ferrat and Siewiera, 2014; Brighton *et al.*, 2017; Sojka *et al.*, 2019; Zhang and Wei, 2021). Furthermore, in the later phase of decidualization, the generated senescent decidual subpopulation was eliminated by uNK cells without affecting mature decidual cells, which are essential for embryo implantation and invasion (Fig. 3a) (Brighton *et al.*, 2017), suggesting that the NK cells play a central role in endometrial cellular senescence.

Recently, the age-dependent depletion of NK cell functions has been demonstrated (Brauning *et al.*, 2022). As a result of this, under the conditions of age-associated alterations, unlike the normal decidualization process, uNK cells lose their ability to eliminate senescent decidual cells and fully senescent ESCs (Deryabin *et al.*, 2020). Thus, this leads to the generation and accumulation of undifferentiated senescent ESCs in the endometrial tissue, which neither proliferate nor have the potential to differentiate into decidual cells. Also, it was shown that the presence of undifferentiated senescent ESCs might negatively affect the emergence of mature and senescent decidual cells subpopulations (Deryabin and Borodkina, 2022). Moreover, the persistence of senescent cells causes prolonged secretion of SASP resulting in an uncontrolled pro-inflammatory milieu during the crucial step of decidualization, which can disturb the secretory cascade of decidual cells and the balance between the pro-inflammatory and anti-inflammatory secretory profiles (Fig. 3b) (Deryabin *et al.*, 2020). SASP produced by senescent ESCs might induce paracrine senescence in the neighbouring normal cells, further reducing the plasticity of endometrial tissue, or might directly impair decidualization of the healthy surroundings, multiplying the negative impact of senescent cells (Griukova *et al.*, 2019; Deryabin and Borodkina, 2022). Moreover, age-associated dysregulation of the cellular senescence phenomenon was also observed in a recent study by our group, which demonstrated the significant upregulation of CDKN2A (p16^INK4A^) in endometrial epithelium of women aged >45 years compared to younger women of <30 years (Loid *et al.*, 2023). Thus, although senescence is one of the vital factors for the initiation of decidualization, age-related cellular changes can impair the senescence-associated network causing an inadequate pro-inflammatory microenvironment that has a detrimental effect on the further pathways involved in decidualization, ECM organization, and hormonal responsiveness of decidual cells, eventually causing failure in embryo implantation (Deryabin and Borodkina, 2022).

#### Endometrial inflammaging and immune tolerance in aged endometrium

##### Endometrial inflammaging

The effect of advanced age causing an aberrant pro-inflammatory response is also referred to as ‘inflammaging’ resulting in hampering the endometrial function (Shirasuna and Iwata, 2017; Hirata *et al.*, 2021). Pertaining to endometrial receptivity, inflammaging can be demonstrated in animal model studies. An *in vitro* bovine endometrial cell model of aged versus young animals revealed an upregulation of pro-inflammatory biomarkers, such as IL1A, interferon regulatory factor 7, and IFNG, involved in inflammatory and interferon signalling pathways contributing to reproductive dysfunction due to advanced age (Tanikawa *et al.*, 2017). The study also demonstrated deregulation in the cell cycle with activation of G2/M DNA Damage Checkpoint Regulation, thus promoting the age-dependent accumulation of DNA damage and cessation of cell proliferation. The imbalance in the cell cycle was associated with elevated secretion of interferons in endometrial cells, resulting in a pro-inflammatory environment in the endometrium, thus making it less responsive to interferon tau (IFNT) leading to the ‘inflammaging’ phenomenon in the bovine endometrium of advanced age (Tanikawa *et al.*, 2017). Additionally, a study comparing younger mares aged 5–7 years with older mares aged ≥15 years showed an age-dependent increased endometrial inflammatory cell infiltration, higher levels of fibrotic changes, and less dense endometrial glands, altogether causing uterine dysfunction, a decreased pregnancy rate, and increased incidence of pregnancy loss (Carnevale and Ginther, 1992). Nevertheless, our knowledge of endometrial inflammaging in humans is less clear.

##### Immune tolerance

As discussed above, an adequate and controlled pro-inflammatory response elicited by CD4+T cells to release Th1 and Th17 subsets of cytokines is essential to promote embryo implantation and trophoblastic invasion (Guerin *et al.*, 2009; Wang *et al.*, 2020a; Wu *et al.*, 2022). In parallel to this, regulatory T cells (Treg) (CD4+ CD25+) also play a vital role in regulating immune tolerance at the implantation site to avoid rejection of the semi-allogeneic embryo during the implantation process (Guerin *et al.*, 2009; Murata *et al.*, 2021). Treg cells target the adaptive immune response by inhibiting proliferation and cytokine production in both CD4+ and CD8+ T cells, thereby supressing the functions of B cells, and antigen-presenting cells such as dendritic cells and macrophages (Guerin *et al.*, 2009). Compared to other immune cell types, the proportion of these cells is lowest during the WOI. At the initiation of decidualization, these cells are accumulated in the endometrium along with the recruitment of other immunological cells and increase during the early pregnancy to mid-gestation to bring about an immunomodulatory reaction with trophoblast cells. Further, the decidual levels of CD4+ CD25+ Treg cells tend to decline as the pregnancy progresses towards delivery (Guerin *et al.*, 2009; Monin *et al.*, 2020; Murata *et al.*, 2021). Similarly, the cytotoxic activity by CD8+T cells is suppressed maximally during the implantation events to create a tolerogenic environment (Shen *et al.*, 2021). However, these cells are found to be prominent during the second and third trimesters of pregnancy, to function in the defence mechanisms against pathogens (Wu *et al.*, 2022).

Although direct evidence of Treg cells or CD8+ T cells affecting the embryo implantation process due to endometrial aging is not available, the decreased populations of CD4+T cells and CD4+Th17 cells, and increased CD8+T cell populations in the endometrium of pre-menopausal women have been reported (Rodriguez-Garcia *et al.*, 2014, 2018). The abundance and functionality of CD4+ T cells gradually decrease with age, eventually compromising their functions such as activation, proliferation, cytokine production, and apoptosis signalling. Thus, it leads to defective immunological synapse formation and cytoskeleton signalling (Garcia and Miller, 2011; Lefebvre and Haynes, 2012). These changes might have started taking place much earlier, even before the perimenopausal period, progressing further with advancing age; however, this needs to be explored in the future. Moreover, the imbalance in the population of CD4+ T cells, CD4+ T17 cells, and CD8+ T cells in human decidua may potentially impact embryo implantation, resulting in an increased rate of spontaneous abortions (Murata *et al.*, 2021).

#### Endometrial fibrosis and aging

The endometrium has been reported to undergo histological changes in response to an imbalance in ovarian hormones caused by advanced age. The ESCs have a considerably lower proliferation rate and become less responsive to decidualization stimuli as age increases (Woods *et al.*, 2017). Moreover, the significant characteristic of aged endometrium is the deposition of collagen in the endometrial stroma with associated tissue fibrosis. Various animal models exhibited age-related increases in uterine fibrosis (Burack *et al.*, 1941; Biggers *et al.*, 1962; Maurer and Foote, 1972; Gosden, 1979; Craig, 1981; Mulholland and Jones, 1993).

Although the mechanism of fibrosis in tissues of older mammals remains elusive, collagen deposition in the endometrium has been reported to be induced by E2 (Mulholland and Jones, 1993; Sharma *et al.*, 2002). This was demonstrated in a study where young ovariectomized rats showed accumulation of collagen after administration of E2 treatment; however, it did not cause an increase in the total uterine collagen, suggesting that the collagen was parallelly degraded as it was synthesized in young rats (Dyer *et al.*, 1980). Similarly, the large bundles of collagen fibrils were present even after the decidualization by aged stromal cells whereas, in young stromal cells, the collagen was observed to have almost disintegrated (Mulholland and Jones, 1993). Thus, collagen deposition in older rats indicates a decline in the potential of collagen degradation as age increases. One of the underlying reasons may be the higher levels of hydroxyproline exhibited by uterine collagen in advanced-age women (Woessner, 1963) and golden hamsters (Rahima and Soderwall, 1977), which affects the degree of crosslinking and thus impairs the degradation potential of collagenases (Mulholland and Jones, 1993). As endometrial aging is associated with inflammation, this can be another important reason for collagen deposition involving various biological functions such as chronic inflammation, oxidative stress, accumulation of senescent cells, caspases, growth factors, etc. (Wynn, 2008). Moreover, the increased level of P4 in rodents at an older age inhibits the action of collagenases in endometrial cells and explant cultures (Jeffrey *et al.*, 1971; Jeffrey, 1981). A micromolar concentration of P4 and glucocorticoids has been shown to impair the synthesis of collagenase in uterine smooth muscle in cell culture (Jeffrey *et al.*, 1990; Mulholland and Jones, 1993). This confirms the notion that the function of collagenases and other proteases decreases with increased age, even in the uterine cells (Maurer and Foote, 1972). The inability of aged endometrium to degrade the collagen also suggests an impairment in the capacity of stromal cells to remodel the ECM organization, which further affects the formation of junctional complexes hampering autocrine and paracrine cell-to-cell signalling events during decidualization (Mulholland and Jones, 1993; Wynn, 2008). Thus, the advanced age and increased fibrosis can be associated with a dysregulation in inflammatory pathways causing pregnancy loss (Hirata *et al.*, 2021). On the contrary, the study by Noci *et al.* (1995) has demonstrated no significant difference in the histology of endometrium with respect to stromal cells and collagen accumulation between younger women <30 years versus older women >40 years. However, older women included in this study group ranged between 40 and 46 years, which may explain the insignificant difference in fibrosis among older women. The correlation of age and collagen accumulation cannot be ruled out in women of more advanced age (>45 years) because most of the notable age-related changes have a negative impact on endometrial biology after 45 years of age. To summarize, aging aggravates collagen deposition along with a decrease in collagen degradation via different mechanisms that ultimately affect various functions, such as ECM organization, decidualization, and uterine senescence, all of which play an important role in female fertility.

#### Alterations in endometrial epithelium and aging

Furthermore, the alterations in endometrial epithelium related to advanced age have been shown using various animal models and even human endometrium. Regarding the animal model studies, the most predominant feature highlighted in aged mice endometrium was variation in number, shape, and distribution of microvilli on the surface epithelium. In aged endometrium, they were of irregular shape, variable in length with an abnormal distribution ranging from tightly clustered to sparsely distributed, giving the appearance of ‘bald’ endometrial cells (Mulholland and Jones, 1993). The cytoplasm was observed to be devoid of organelles with tufts of fine filaments composed of proteins from collapsed microvilli. Intra-epithelial leukocytes, primarily lymphocytes, were observed to be elevated in old rats compared to younger rats (Mulholland and Jones, 1993).

Moreover, the endometrial laminar and glandular epithelium contain ciliated cells, which are believed to aid in the transportation of glandular secretions. They also help the gamete and embryo to move along the uterine lining. However, their exact role in this context remains unclear (Newman *et al.*, 2023). The populations of ciliated epithelial cells in the endometrium exhibit significant variation in numbers during the menstrual cycle and they also have distinct transcriptomic profiles as compared to other endometrial cell types (Wang *et al.*, 2020b; Garcia-Alonso *et al.*, 2021; Loid *et al.*, 2023). It is unclear whether a higher quantity of ciliated epithelial cells creates a favourable environment or, conversely, hinders trophoblast invasion. Also, as multiciliated cells possess sensory functions through hormone and IL receptors on their surface (Nutu *et al.*, 2009; Shao *et al.*, 2009), compromised cilial development may interfere with embryo-uterine communication.

### Effect on endometrial aging: molecular aspects

Several genomic studies using animal models have identified the impact of aging on endometrial receptivity and implantation. The study in the murine model revealed upregulation of the pro-inflammatory cytokine *il17rb*, and chemokines such as *cxcl12*, and *cxcl14* in aged versus young wild-type mice (Kawamura *et al.*, 2021). Additionally, altered expression of genes associated with mitotic division, angiogenesis, cell migration, immune response, and inflammation was observed in aged mice (Kawamura *et al.*, 2021). Another *in vitro* transcriptome study in mice endometrium showed delayed decidualization of stromal cells and impairment in the progression of decidual differentiation with significant downregulation of key regulatory genes like *Prl8a2 (Dtprp), Bmp2, Hand2, Hoxa10, Nr2f2 (Coup-tf II), Igfbp5, Sfrp5, Ltf, Muc1 and Cdh1* in aged females (Woods *et al.*, 2017).

Moreover, the recent *in-vitro* candidate gene study in human primary stromal cells (PSC) reported an age-dependent decrease in the proliferative capacity of stromal cells with a significant decrease in mRNA and protein expression of BMP3 and STAT3 in women aged ≥36 years (Berdiaki *et al.*, 2022). Furthermore, *in vitro* induction of decidualization of PSC in aged women showed significant mRNA downregulation of the decidualization markers *IGFBP1* and *PRL*, indicating that the age-related dysfunction in stromal cell proliferation can potentially eventually affect decidualization, endometrial receptivity, endometrial thickness, and embryo implantation (Berdiaki *et al.*, 2022).

Based on the available literature, there are very limited data on endometrial aging and its effect on receptivity in humans using a genome-wide approach. Intracellular and molecular interactions in mid-secretory endometrium of healthy women and infertile patients have been studied using advanced omics technologies (Messaoudi *et al.*, 2019; Ruiz-Alonso *et al.*, 2021). These studies have led to the development of molecular diagnostic tools for the assessment of endometrial receptivity and to pinpoint the exact day for embryo transfer in patients suffering from recurrent implantation failure. However, in these studies, the factor of ‘advanced age’ has not been explored; thus, the molecular mechanism of endometrial aging and its impact on receptivity potential has not been well established.

Recently, *in silico* tools have been used to retrospectively analyse the published gene expression datasets based on the ‘age’ factor varying from 23 to 43 years (Devesa-Peiro *et al.*, 2022). The study revealed a total of 5778 differentially expressed age-related genes involved in the dysregulation of biological functions, such as inhibition of epithelial cell proliferation, vascular endothelial growth factor signalling, angiogenesis, insulin signalling, and aging hallmark processes, such as cell cycle arrest and telomer protection, among women 35 years or older (Devesa-Peiro *et al.*, 2022). A recent study by our group demonstrated that, in addition to general cell cycle and immune pathways dysregulated in women over 45 years, a potent senescence marker CDKN2A (also known as p16^INK4A^) is significantly upregulated in the endometrial epithelium of advanced age women. The accumulation of p16-positive cells may lead to specific changes in endometrial epithelial development through interference with extracellular signal-regulated kinase (ERK), Notch and Hedgehog pathways (Loid *et al.*, 2023).

Additionally, as reported by Devesa-Peiro *et al* (2022), the majority of differentially expressed genes were associated with ciliary processes, cilia motility, and ciliogenesis. Similar findings were obtained by Loid *et al.* (2023) wherein a large number of cilia-associated genes was up-regulated in endometrial samples corresponding to the WOI among advanced age women (>45 years) compared to younger women (<30 years). The study also identified a larger population of ciliated epithelial cells in women of advanced age (Loid *et al.*, 2023). Likewise, age-related differences have also been reported in a recent study performed on microRNAs. According to this study, the levels of miR-449c.1 and its precursor were downregulated in the mid-secretory endometrium of a 49-year-old woman compared to a 34-year-old woman of reproductive age. The miR-449c.1 belongs to the miR-34/449 family, which is involved in the regulation of cilia formation that is a prerequisite for the establishment of endometrial receptivity (Naydenov *et al.*, 2022). Endometrial ciliated cells are transcriptionally distinct epithelial cells with varying numbers of cell populations across the menstrual cycle (Wang *et al.*, 2020b; Garcia-Alonso *et al.*, 2021), and therefore, their functions and optimal number need to be investigated further with respect to endometrial receptivity and chronological aging.

Embryo implantation is a result of the successful dialogue between receptive endometrium and the competent embryo. In other words, endometrial receptivity is also modulated by the embryo itself. The interactions between endometrium and embryo have been explained by the concept of exosome biogenesis (Ntostis *et al.*, 2021). Extracellular exosomes secreted by the receptive endometrium, as well as by the competent embryo, encapsulate essential signals for the implantation process. At a younger age and under a favourable microenvironment created by steroid hormones, anti-apoptosis, and energy generation pathways, these exosomes exchange signals, which induce cell adhesion and cell migration, to achieve synchronized embryo-endometrial co-ordination. Trophectoderm derived from the embryos of younger women showed upregulation of genes associated with extracellular exosome biogenesis, reduction of oxidative stress, mitochondrial ATP synthesis, and cholesterol biosynthesis compared to women of advanced age. Thus, in the implantation process, exosome-related genes derived from the trophectoderm are altered in women of advanced age compared to younger women, leading to unsuccessful embryo-endometrial competency (Ntostis *et al.*, 2021). Moreover, maternal age-related asynchrony between embryo and endometrium has also been demonstrated in the stimulated IVF cycles wherein the 50% chance of asynchrony in women younger than 35 years of age is elevated to 65% in women of advanced age (Shapiro *et al.*, 2016). Still, although recent studies have supported the hypothesis that age affects endometrial function, the exact mechanism(s) and integrated associated factors involved needs to be further elucidated to generate substantial evidence.

## Endometrial epigenetic aging

The term aging is generally applied to the chronological age of the individual. However, the concept of biological age has been introduced recently, according to which each individual, or more specifically each tissue type, has a biological age that can differ from the chronological age (Franceschi *et al.*, 2018). The biological age of an individual can be assessed by virtue of the ‘epigenetic clock’ or ‘methylation age’ (Horvath, 2013) as well as the ‘telomer clock’ (Shammas, 2011). However, the literature associated with the biological age of the endometrium is more prevalently reported in terms of epigenetic alterations.

Epigenetics has a vital role in several biological processes including all crucial stages of reproduction (Retis-Resendiz *et al.*, 2021). DNA methylation is a widely studied aspect of epigenetic regulation that shows age-related alterations characterized by hypo- or hypermethylation of CpG sites in the genes (Horvath, 2013). The age-associated deviation in the methylation pattern varies with tissue type, which enables us to define the biological age of that particular tissue. Based on this approach, for the first time, ‘Horvath’s epigenetic clock’ was developed in multiple tissues, which aimed to predict the biological age of the tissues and may even predict the attributed risk for disorders such as cancer (Horvath, 2013). Horvath’s epigenetic clock, which was based on the DNA methylation status of 353 CpG sites and was tested across 51 human tissues, however, showed a marginal correlation of chronological age with endometrial epigenetic age (*R* = 0.55). After the Horvath’s clock, a ‘DNA methylation Pheno-Age clock’ was developed using age-associated significant biomarkers from whole blood to predict the phenotypic age and then correlated with multi-tissues and different cell types (Levine *et al.*, 2018). However, the correlation of chronological age and epigenetic age of human endometrium was not accurately predicted by the Pheno-Age clock (*R* = 0.39) (Levine *et al.*, 2018; Deryabin and Borodkina, 2023). Another predictor of methylation age, with improved precision, was developed called ‘Zhang clock’ which was primarily based on blood but was tested across non-blood tissues (Zhang *et al.*, 2019). The Zhang clock showed higher accuracy in prediction of the epigenetic age of endometrium (*R* = 0.77) than the original Horvath’s clock. Recently, the ‘AltumAge’ epigenetic clock (de Lima Camillo *et al.*, 2022) was recalibrated in multi-tissues with higher prediction accuracy using machine learning models (Vijayakumar and Cho, 2022), showing improved performance, for the correlation of chronological age and endometrial age (*R* = 0.78). Thus, the last two epigenetic predictors; Zhang clock and ‘AltumAge’ epigenetic clock, as well as Horvath’s clock after the menstrual phase correction were more accurate and precise predictor tools for endometrial age, with decreased mean absolute error (Deryabin and Borodkina, 2023).

The endometrium is a very dynamic tissue that undergoes cellular and biochemical alterations during different phases of the menstrual cycle associated with transcriptomic alterations regulating the molecular milieu (Wang *et al.*, 2020b). The differential gene expression across the menstrual cycle is regulated temporally and spatially to some extent by epigenetic mechanisms such as DNA methylation (Retis-Resendiz *et al.*, 2021). The DNA methylation profile in the endometrium of healthy fertile women changes as it shifts from a pre-receptive to receptive endometrium and correlates with alterations in the gene expression profile across the menstrual cycle (Houshdaran *et al.*, 2014; Kukushkina *et al.*, 2017), having a unique methylation profile during the WOI (Kukushkina *et al.*, 2017). This suggests that age can influence the endometrial DNA methylation pattern based on the menstrual phase. This can cause significant changes in the methylation levels of CpG sites of the epigenetic clock and eventually deviate the biological age of the endometrium (Olesen *et al.*, 2018). This hypothesis was further confirmed by evaluation of endometrial age by Horvath’s epigenetic clock using menstrual phase correction in healthy women at the mid-secretory phase having a regular menstrual cycle. After the menstrual phase correction, Horvath’s clock showed an improved correlation between chronological age and endometrial epigenetic age (from *R* = 0.55 versus *R* = 0.80), with a reduction in mean absolute error from 11 to 4.4 years. Thus, the endometrium collected at the specific time-point was more accurately synchronized with the chronological age, with a higher correlation (Olesen *et al.*, 2018). This correlation was also inferred to be constant, even for the second endometrial biopsy in the consecutive menstrual cycle. Thus, the approach of standardized sampling time in combination with the highly precise epigenetic clock can be used to adjust the epigenetic clock for the accurate prediction of endometrial age (Deryabin and Borodkina, 2023).

Since an aberrant endometrial DNA methylation profile has been correlated with disorders such as endometriosis (Xue *et al.*, 2007; Saare *et al.*, 2016) and cancer (Multinu *et al.*, 2020), biological age can also be used as a predictive biomarker for pathology progression. A recent study showed that the methylation pattern of the *PTENP1* pseudogene (regulator of tumour suppressor gene PTEN) in the human endometrium is also age-dependent (Kovalenko *et al.*, 2021). The level of methylation and associated expression of the endometrial *PTENP1* pseudogene was elevated in women aged 45 years or older. Interestingly, the age-specific alteration in methylation and subsequent expression of *PTENP1* pseudogene was hypothesized to serve as a protective mechanism against endometrial malignancies among older women heading towards menopause (Kovalenko *et al.*, 2021).

As postulated in a recent review, epigenetic alterations can be reversed by modifications in environmental and dietary factors, administration of drugs targeting epigenetic pathways and genetic reprogramming using OSKM transcriptional factors (OCT4, SOX2, KLF4, and C-MYC) (Deryabin and Borodkina, 2023). Implementation of these approaches can be used to slow down or reverse the affected epigenetic clock and can be considered to diminish the age-related impairment in the endometrium (Deryabin and Borodkina, 2023).

## Human models to study the clinical outcome of endometrial aging

### Oocyte donation cycles and the effect of aging on endometrial receptivity

Advanced age is one of the major obstacles in achieving a clinical pregnancy and live birth. The gathered evidence has proven that advanced age can affect oocyte quantity, oocyte quality and, eventually, embryo quality, which can be accountable for pregnancy loss, decreased implantation rate, and complications in pregnancy outcome (Cimadomo *et al.*, 2018; Zhang *et al.*, 2020). This can be resolved to some extent with the transfer of embryos generated by fertilizing donor oocytes retrieved from younger women (Seshadri *et al.*, 2021). The success of oocyte donation can be influenced by various factors, for example, the age of the oocyte donor and recipient, clinical indication for oocyte donation, embryo quality, endometrial thickness and blood flow in the recipient (Moomjy *et al.*, 1999; Noyes *et al.*, 2001; Huang *et al.*, 2008; Braga *et al.*, 2020). Although the donor’s age can be controlled in oocyte donation, the effect of the recipient’s age on the endometrial receptivity can be a critical factor in the success of oocyte donation cycles and thus cannot be overlooked. In human studies, oocyte donation may therefore serve as a good model to evaluate the endometrial-associated factors independent of oocyte quality in women of advanced age.

**Figure 4.**
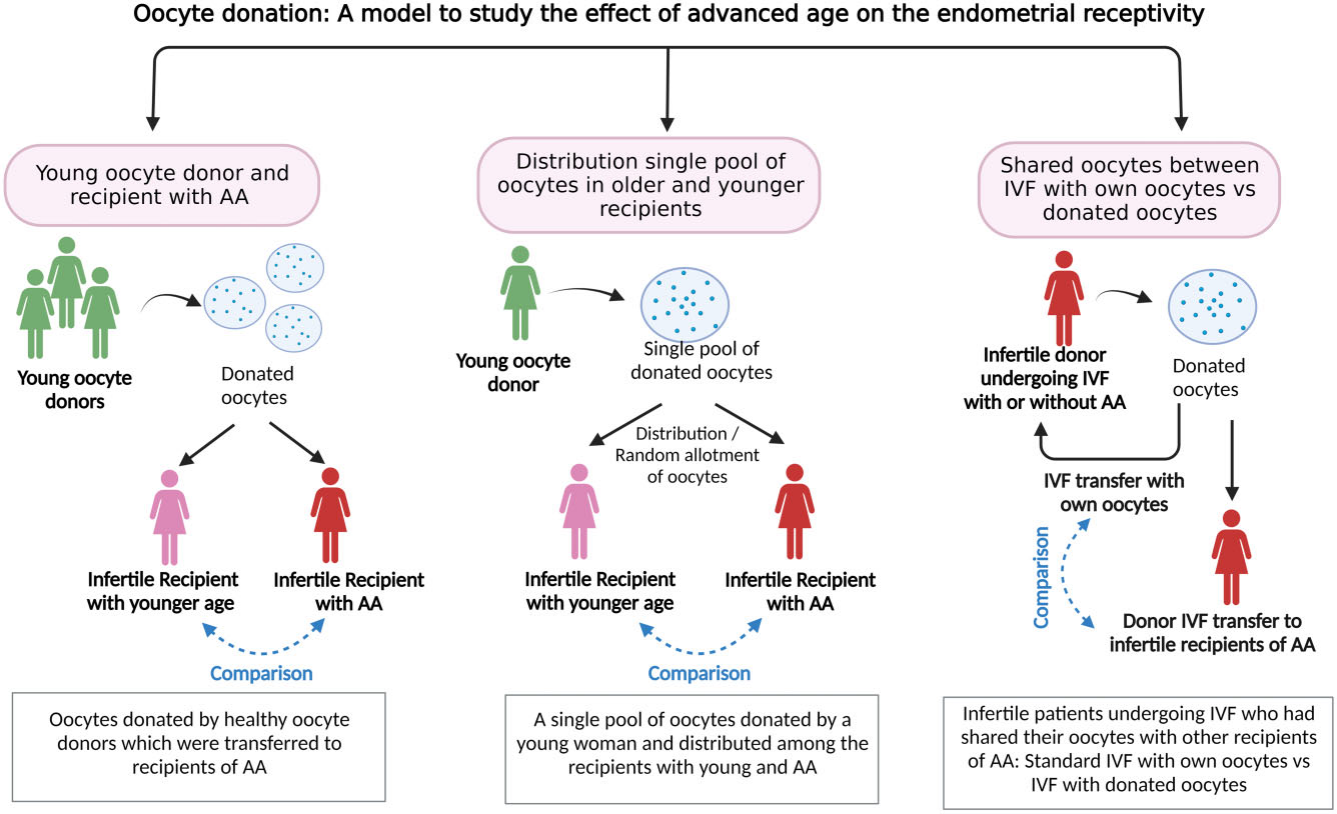
**Oocyte donation: a model to study recipient aging and her endometrial receptivity.** The reported studies have been divided into three arms: oocytes donated by healthy oocyte donors which were transferred to younger recipients or those of advanced age (left); a single pool of oocytes donated by a young woman and distributed among young and advanced age recipients (middle); and infertile patients undergoing IVF who had shared their oocytes with other recipients of advanced age: standard IVF with own oocytes versus IVF with donated oocytes (right). AA, advance age. Created with BioRender.com (https://biorender.com/).

The respective studies can be divided into three main groups based on their inclusion criteria and study design (Fig. 4 and Table 1): studies including healthy, younger oocyte donors and recipients with advanced age to deduce the effect of recipient’s age on endometrial receptivity; studies in which the oocytes donated from single young and healthy woman were distributed amongst the recipients of younger and advanced age; and studies undertaken wherein the infertile patients undergoing IVF had shared their oocytes with other recipients of advanced age, thus indicating the effect of age on endometrial receptivity between IVF cycles using own oocyte and donated oocytes using the same pool of oocytes retrieved from same women.

**Table 1.** Summary of studies performed in oocyte donation IVF cycles to determine the impact of advanced age on endometrial receptivity.

Study design	**Reference studies that suggest AA** ^1^ **has an impact on ER**^2^		Reference studies that suggest AA has no impact on ER
	First author, year	Detailed conclusion if any	First author, year
Oocytes donated by healthy oocyte donors which were transferred to recipients of AA	Abdalla *et al.* (1990)	–	Balmaceda *et al.* (1994)
	Meldrum (1993)	The impact of age on ER can be reversed	Sauer *et al.* (1994)
	Weckstein *et al.* (1993)	The impact of age on ER can be reversed	Legro *et al.* (1995)
	Yaron *et al.* (1998)	–	Remohi *et al.* (1997)
	Moomjy *et al.* (1999)	Impact of age on IR^3^ but not on PR^4^ and miscarriage rate	Paulson *et al.* (1997)
	Toner *et al.* (2002)	Impact of age on ER after 40 years of age	Mirkin *et al.* (2003)
	Soares *et al.* (2005)	Impact of age on ER after 45 years of age	Wang *et al.* (2012)
	Yeh *et al.* (2014)	Impact of age on ER after 45 years of age	–
A single pool of oocytes donated by a young woman and distributed among the recipients with young and AA	Cano *et al.* (1995)	–	Navot *et al.* (1994)
	Harris *et al.* (2002)	AA has a negative impact on the live birth rate but not on PR	Noyes *et al.* (2001)
Infertile patients undergoing IVF who had shared their oocytes with other recipients of advanced age: standard IVF with own oocytes versus IVF with donated oocytes	Levran *et al.* (1991)	–	Navot *et al.* (1991)
	Check *et al.* (1993a)	–	Check *et al.* (1994)
	Borini *et al.* (1996)	–	
	Braga *et al.* (2020)	–	–

1 AA: advanced age.

2 ER: endometrial receptivity.

3 IR: implantation rate.

4 PR: pregnancy rate.

#### Young oocyte donors and recipients with advanced age

In this group, young oocyte donors had donated their oocytes to the recipients of advanced age (Table 1 and Fig. 4; Supplementary Table S1). Several studies in this group have reported that the recipient’s age had no impact on endometrial receptivity (Balmaceda *et al.*, 1994; Sauer *et al.*, 1994; Legro *et al.*, 1995; Paulson *et al.*, 1997; Mirkin *et al.*, 2003; Wang *et al.*, 2012). The studies, including both fresh and frozen embryo transfer cycles with the recipient age groups ranging from 23 to 54 years, revealed no significant differences in endometrial thickness, pregnancy rate, miscarriage rate, and implantation rate among the different age groups of recipients (Balmaceda *et al.*, 1994; Mirkin *et al.*, 2003). However, this result applies only to patients having endometrial thickness ≥8 mm because the pregnancy rate was observed to drop from 45% to 17% in the recipients with an endometrial thickness of 4–8 mm (Mirkin *et al.*, 2003). Comparable findings were reported in the studies where the recipient’s age had no impact even on the cumulative pregnancy rate and cumulative live birth rate after four consecutive cycles (Paulson *et al.*, 1997; Remohi *et al.*, 1997).

On the other hand, a decreased pregnancy rate was observed with increasing age of recipients (Yaron *et al.*, 1998). Another study demonstrated a drastic decline in endometrial receptivity, with a reduction in the delivery rate from 46% to 21% and an elevation of the abortion rate from 17% to 41% as the recipient’s age increased over 40 years (Meldrum, 1993). This study postulated that the distribution of ER in endometrium decreases with age, which further causes the downregulation of PR leading to impairment in the endometrial microenvironment, making it unfavourable for implantation. The endometrial receptivity was restored, as shown by an increase in the delivery rate from 21% to 54%, by administering a higher dose of exogenous P4 from 50 to 100 mg in advanced-age recipients (Meldrum, 1993). Similarly, the implementation of additional P4 improved the clinical pregnancy rate significantly from 24% to 66% in recipients aged 40 years and above (Weckstein *et al.*, 1993). However, one study (Abdalla *et al.*, 1990) reported a steady decline in pregnancy rate from 50% to 9.7% among the group of recipients aged 25–29 and 45–49 years, respectively, even after prescribing a sufficiently higher dose of P4. Still, these studies collectively highlight that the aged endometrium with altered uterine vasculature and dysregulated PR can potentially restore the receptive potential upon supplementation of additional P4.

Furthermore, an analysis of national data on oocyte donation IVF cycles of the US registry in 2002 (Toner *et al.*, 2002) revealed that the implantation rate, pregnancy rate, and delivery rate were stable for the recipient’s age group ranging from 25 to 40 years, whereas a decline in fecundity was reported in women of advanced age, in their late 40s. In 2014 (Yeh *et al.*, 2014), the US national registry inferred a cut-off of 45 years for the decline in female fertility; more specifically it showed that the age group of 45–49 years can be designated as a transitional period for recipients, which indicates the gradual decrease in pregnancy success. A similar cut-off point of 45 years that affects female fertility was found in a study with a large sample size of 3089 donation cycles (Soares *et al.*, 2005). However, a clinical diagnosis of the recipient may also determine the effect of the recipient’s age on endometrial receptivity. For example, a direct correlation of a decline in implantation rate with increasing age has been found in recipients with tubal disease (Moomjy *et al.*, 1999).

#### A single pool of oocytes donated by a young woman and distributed among young and advanced-age recipients

Furthermore, to increase the specificity and precision, the oocytes from a single pool donated by a young and healthy woman were distributed among recipients of advanced age and of younger ages (Table 1 and Fig. 4; Supplementary Table S2). In such types of studies, the effect of endometrial aging can be assessed precisely as the parameter of oocyte quality remains constant. One such study reported no significant difference in implantation rate and delivery rate among the recipients aged <40 and ≥40 years, for an even and random distribution of oocytes (Navot *et al.*, 1994). The study by Noyes *et al.* (2001) had similar findings of showing no effect of the recipient’s age on the live birth rate; however, the live birth rate significantly lowered as the endometrial thickness was <8 mm.

In another study, the oocytes donated by fertile as well as by infertile women undergoing IVF were divided evenly between younger and older recipients, with an age limit of 40 years (Cano *et al.*, 1995). Although the implantation rate between the two groups of recipients did not show any significant difference, the abortion rate was significantly higher in older recipients owing to impaired uterine vascularization and late secretion of E2 and P4 by the placenta, reducing its functional efficacy.

As the factors associated with oocyte donors can be controlled, the importance of the careful selection of donors to improve the success rate of IVF needs to be considered (Harris *et al.*, 2002). Moreover, it has been observed that the donor’s parity and the history of miscarriage can have a causal effect on the probability of pregnancy in their corresponding recipients (Harris *et al.*, 2002).

#### Infertile patients undergoing IVF who had shared their oocytes with other recipients of advanced age: standard IVF with own oocytes versus IVF with donated oocytes

Some studies have been undertaken wherein the infertile patients undergoing IVF shared their oocytes with recipients of advanced age, thus comparing standard IVF cycles using women’s own oocytes and IVF cycles with donated oocytes (Table 1 and Fig. 4; Supplementary Table S3). Using this study design, both oocyte aging and endometrial aging can be studied simultaneously. Such studies, wherein the donors and advanced-aged recipients had received the unselected oocytes from the same cohort, revealed a similar clinical pregnancy rate in recipients and donors irrespective of age (Navot *et al.*, 1991; Check *et al.*, 1994).

On the contrary, other reports with a similar study design have shown an age-related negative impact on endometrial aging. These reports suggest a decline in endometrial receptivity among older recipients compared with younger donors who have undergone the embryo transfer with their own oocyte (Levran *et al.*, 1991; Check *et al.*, 1993a; Borini *et al.*, 1996; Braga *et al.*, 2020). Overall, the literature on treatment options for oocyte donation in women of advanced age showed very ambiguous inferences based on endometrial aging.

## Anti-aging therapies and endometrial receptivity

The treatment option for endometrial aging in patients with advanced age is to treat the cellular senescence process associated with endometrial aging. Scientific advances have led to the development of drug therapies which can target senescent cells and prevent their detrimental effect causing pathogenic conditions, including age-related diseases. Such drugs are referred to as ‘senolytics’, ‘senomorphics’, or ‘senostatics’, which cause apoptosis of the senescent cells or inhibit the function of senescent cells and their secretions in terms of SASP (Deryabin *et al.*, 2020; Secomandi *et al.*, 2022). There are several senolytic drugs, using different interventions or modes of action, to target the senescent cells, such as attenuation of the pro-survival pathway, anti-apoptotic proteins, targeting cellular metabolism pathway of senescent cells, and stimulation of the immune system for the targeted clearance of senescent cells (Secomandi *et al.*, 2022).

Various senolytic therapies have been used in animal models for several age-associated pathological conditions, such as idiopathic pulmonary fibrosis, kidney diseases, and atherosclerosis as well as ovarian dysfunction, and can even restore the pregnancy rate and oocyte competence (Sugiyama *et al.*, 2015; Bertoldo *et al.*, 2020; Secomandi *et al.*, 2022). However, the implementation of senolytic therapies to improve endometrial receptivity is extremely challenging. As discussed above, the phenomenon of cellular senescence is essential in the initial phase of decidualization to elicit the necessary pro-inflammatory response to create a favourable microenvironment and to open the temporal window for successful implantation. Aging may cause inability of the immune system to eliminate senescent ESCs, which may interfere with the inflammatory balance during the decidualization process (Brighton *et al.*, 2017). Thus, advanced age can alter the normal senescence phenomenon that is essential for endometrial receptivity. Senolytic therapy targeted to the senescent cells can affect the pre-senescent ESC and senescent decidualized cells; thus, there is a possibility of destroying either or both of the cellular populations, which could have been contributing to endometrial receptivity (Deryabin *et al.*, 2020; Secomandi *et al.*, 2022). To resolve this problem, the senolytic agent dasatinib, a selective tyrosine kinase receptor inhibitor that inhibits the cellular apoptosis resistance pathway causing an apoptotic effect on senescent cells, has been investigated. A study with ESC culture was conducted involving the targeted elimination of pre-senescent ESCs using dasatinib treatment, which resulted in a significant reduction in pro-inflammatory secretion during decidualization (Brighton *et al.*, 2017). Furthermore, a combination of dasatinib and quercin was used as the inhibitor of cellular pro-survival pathways in the murine uterus *in vitro*, which showed upregulation of the p53 pathway and suggested an anti-fibrotic uterine effect. However, the results could not be replicated *in vivo* (Cavalcante *et al.*, 2020). Additionally, senomorphic agents such as rapamycin or resveratrol, used for the disruption of undifferentiated senescence cells, showed impairment of the decidualization process (Brighton *et al.*, 2017; Ochiai *et al.*, 2019). Resveratrol treatment induces dysfunction of a retinoic acid signalling pathway in decidualized ESCs, which has a detrimental effect on decidualization causing a reduction in implantation rate and increasing the risk of miscarriage when used *in vivo* (Ochiai *et al.*, 2019). At the same time, a recent study revealed that pre-treatment of senescent ESCs with senomorphic agents such as metformin or rapamycin might improve further decidualization and blastocyst implantation, at least *in vitro* (Deryabin and Borodkina, 2022). It is likely that negative or beneficial outcomes of senomorphics might be related to the phase of the menstrual cycle. Indeed, if resveratrol is added during the WOI, it would further negatively affect embryo implantation (Brighton *et al.*, 2017; Ochiai *et al.*, 2019). However, if resveratrol supplementation is restricted to the proliferative phase, it would not adversely impact embryo implantation or ESCs decidualization (Kuroda *et al.*, 2020). Therefore, the use of senolytic therapy to improve decidualization and endometrial receptivity needs to be considered with extreme caution. For this purpose, the factors such as time period, dosage, phase of the menstrual cycle, adverse effects and potential risk for generation of inappropriate inflammatory response should be critically evaluated by means of precise experimental studies and appropriately designed clinical trials.

## Conclusion

Advanced age of the woman can be one of the significant risk factors for the impaired cellular senescence phenomenon and defective endometrial receptivity, which may contribute to infertility. Published studies using animal models have uncovered the potential risk of tissue fibrosis with changes in the tissue histology, dysregulation of the decidualization process, and immune and inflammatory imbalance, causing deterioration of endometrial receptivity as age increases. However, limited publications in this area indicate that the effect of increasing age, specifically on human endometrium, has been inadvertently underestimated. In the past two decades, the molecular mechanism, a network of biological pathways, and biomarkers of endometrial receptivity have been elucidated in the literature with the main focus being on the development of diagnostic tools for its assessment (Ruiz-Alonso *et al.*, 2021). However, information on the correlation between advanced age and the molecular aspects of endometrial receptivity in these studies is mostly lacking. A recent study based on transcriptome datasets of endometrium in different reproductive age groups revealed that endometrial gene expression was altered after the age of 35 years (Devesa-Peiro *et al.*, 2022); although the design of this study was retrospective, it is the only published study that provides endometrial data determining the role of maternal age using the whole-genome approach. Owing to the paucity of evidence in this domain, it is difficult to draw a concise conclusion pertaining to the cut-off age for the physiological initiation of endometrial aging. Therefore, there is a need for prospective research studies with appropriate designs to detect the influence of age on various aspects of endometrial functioning. Such studies can be aimed at detecting the age-related distribution of hormone receptors and immunological biomarkers in human endometrium, and to evaluate endometrial profiling along with cellular heterogeneity in the ciliated epithelial cell populations among older versus younger women to understand the exact mechanism(s) affecting endometrial receptivity. Additionally, investigating the correlation between endometrial thickness and ultrasound pattern with advanced age using clinical trials would further contribute to the clinical implications associated with pregnancy outcomes. Moreover, future studies can be conducted considering the hypothesis of establishing a personalized age threshold at which the endometrium starts displaying adverse effects of aging.

Furthermore, oocyte donation is one of the best models to study the effect of age on the recipient’s endometrium by using the oocytes from younger and healthy donors. Although different study designs and variables have been used in the literature on oocyte donation cycles, the resulting data have contradictory inferences about the impact of advanced age on implantation or pregnancy rate in recipients. In the future, randomized controlled clinical studies evaluating the impact of using oocytes from younger donors on the endometrium of advanced-aged recipients, using clinical pregnancy rate and live birth rate as the main outcome measures, would be promising. Looking at the perspective of treatment options to restore endometrial receptivity in older women, several senolytic therapies have been used in animal models (Secomandi *et al.*, 2022). However, because cellular senescence is essential during decidualization, implementing these senolytic agents to restore endometrial receptivity without hampering the decidualization is extremely challenging.

Thus, in this review, we have attempted to cover most of the aspects related to endometrial receptivity that can be influenced by age and that would exert an impact on the implantation of the embryo. This review was restricted to conception and the implantation process, and the subsequent features related to miscarriage and pregnancy-associated complications have not been covered.

In summary, although the exact mechanisms involved in the effects of advanced age on endometrial receptivity are still elusive, the outcomes of recent publications are suggestive of the negative effect of aging on endometrial biology. However, cumulative scientific literature indicates a dearth of research studies conducted in this area, that results in a knowledge gap concerning the underlying causes and associated effects. To validate these findings, future research is needed, focusing on the molecular mechanisms underlying the effect of age on cellular senescence, cellular compositions, and transcriptomic changes with regard to endometrial receptivity. This research can utilize advanced techniques, such as single-cell sequencing, to gain a comprehensive and deeper understanding of these processes. Additionally, there is a need for continued research to support the development of treatment options that address the age-related decline in endometrial receptivity. Exploring the use of senolytic agents, which can selectively eliminate senescent cells, to restore endometrial receptivity without compromising the decidualization process would be a promising avenue to investigate. In parallel, in the future, studying the model of oocyte donation cycles with an unbiased study design using clinical trial protocols would be constructive in order to understand the direct clinical implications of endometrial aging on pregnancy outcomes.

## Supplementary Material

dmad019_Supplementary_Data

## Data Availability

No new data were generated or analysed in support of this research.
