# Supplementary material for: Endometrial receptivity in women of advanced age: an underrated factor in infertility
Source: Hum Reprod Update. 2023 Jul 19;29(6):773–93. doi: 10.1093/humupd/dmad019 (PMC10628506; doi:10.1093/humupd/dmad019)
Supplement: dmad019_Supplementary_Data [file dmad019_supplementary_data.zip › dmad019_Supplementary_Data/Supplementary_table_S1 final.docx]

**Supplementary Table SI:** Oocytes donated by healthy oocyte donors which were transferred to recipients of advanced age (AA).

| **REF** | **Study population** | **Outcome measure** | **Donor age groups/**  **range/**  **Mean age**  **(years)** | **Recipient’s age**  **(years)** | **The outcome in the recipients**  **PR/IR/AR/DR/LBR (%)** | **Conclusion** | **Negative impact on ER**  **Yes/No** |
| --- | --- | --- | --- | --- | --- | --- | --- |
| Balmaceda et al., 1994 | 258 cycles of oocyte donation were performed in 182 recipients | PR, IR, and AR | Age groups:  <30,  31-35  36-39 | ≤30 | PR: 37.5, IR: 13,  AR: 16.7 | No influence of recipients’ age on pregnancy, implantation and abortion rates | No |
|  |  |  |  | 31-35 | PR: 53.6, IR: 20.6,  AR: 13.3 |  |  |
|  |  |  |  | 36-40 | PR: 43, IR: 17.3,  AR: 11.1 |  |  |
|  |  |  |  | 41-45 | PR: 40, IR: 14,  AR: 13.6 |  |  |
|  |  |  |  | 46-53 | PR: 53.8, IR: 12.2,  AR: 7.1 |  |  |
| Legro et al., 1995 | 307 consecutive donor oocyte cycles | Ongoing or delivered PR/ET and MR | Range: 21-42  Mean: 32.6 | ≤42 | Ongoing or delivered PR/ET 30.2  MR: 9.8 | Recipients’ age does not affect the oocyte donation cycle outcome and MR | No |
|  |  |  | Range: 21-42  Mean: 31.5 | >42 | Ongoing or delivered PR/ET 30.6  MR: 16.3 |  |  |
| Mirkin et al., 2003 | A total 257 fresh ET and 96 Cryo-ET cycles were studied in 231 recipients | CPR/ET, IR, LBR, and miscarriage rate | 20-33 | 25-54 Fresh ET | CPR/ET: 48,  IR: 22, MR: 19 | Recipient’s age had no statistically significant differences in CPR, IR, MR | No |
|  |  |  |  | 25-54 Cryo-ET | CPR/ET: 34,  IR: 17, MR: 12 |  |  |
| Paulson et al., 1997 | 418 fresh ET cycles among 276 recipients | Overall CPR, Cu-PR/4ET, overall DR, Cu-DR/4ET | <36 | <30  30-39  40-49  50-59 | Overall CPR: 36.2  Cu-PR/4ET: 87.9  Overall DR: 29.3  Cu-DR/4ET: 86.1 | No significant differences within all the age groups of recipients | No |
| Remohi et al., 1997 | 397 recipients undergoing a total of 627 ET cycles | PR/ET, DR, Cu-PR/4ET, LBR | 18-35 | ≤30  31-35  36-40  41-45  ≥46 | PR/ET:53.4  DR: 42.6  Cu-PR/4ET: 94.8  Cu-LBR/4ET:88.7 | No significant differences in all age groups of recipients | No |
| Sauer et al., 1994 | 300 oocyte donation cycles and 192 recipient couples | Cu-PR, IR, DR | Under 35 | <30 | Cu-PR: 50,  IR: 11.1, DR: 32 | No impact of age of the recipient on ER | No |
|  |  |  |  | 30 to 39 | Cu-PR: 47.5,  IR: 15.1, DR: 31.3 |  |  |
|  |  |  |  | 40 to 49 | Cu-PR: 52.3,  IR: 15.2, DR: 29.5 |  |  |
|  |  |  |  | 50 to 59 | Cu-PR: 55.6,  IR: 17.9, DR: 37.5 |  |  |
| Wang et al., 2012 | 3,889 fresh ET cycles | PR and LBR | Age groups: <30  30-34  35-39  ≥40 | <35 | PR: 34.3,  LBR: 25.6 | No significant differences in PR and LBR as per recipient’s age | No |
|  |  |  |  | 35–39 | PR: 33.7,  LBR: 25.4 |  |  |
|  |  |  |  | 40–44 | PR: 33.7,  LBR: 25.6 |  |  |
|  |  |  |  | ≥45 | PR: 36.2,  LBR: 25.8 |  |  |
| Abdalla et al., 1990 | 82 IVF patients had 100 cycles of oocyte donation from 68 donors | PR | 22-38 | 25-29 | PR: 50 | The age of recipient significantly affected the PR | Yes |
|  |  |  |  | 30-34 | PR: 36 |  |  |
|  |  |  |  | 35-39 | PR: 28.5 |  |  |
|  |  |  |  | 40-44 | PR: 18.2 |  |  |
|  |  |  |  | 45-49 | PR: 9.7 |  |  |
| Meldrum, 1993 | Effect of recipients’ age on oocyte donation and the effect of different doses of P4 on ER | Ongoing-delivered PR, AR, and the effect of exogenous P4 dose | <35 | Younger group <40 | Ongoing-delivered PR: 46, AR: 17 | The decline in PR was corrected by doubling the P4 dose | Yes, however, can be reversed |
|  |  |  |  | Older group >40 | Ongoing-delivered PR: 21, AR: 41  PR50P^[[1]](#footnote-1)^#:21  PR100P^[[2]](#footnote-2)^φ: 54 |  |  |
| Moomjy et al., 1999 | 370 oocyte recipients undergoing fresh ET | PR, DR, MR, and IR | 21-35 | ≤ 34 | PR: 58, MR: 8,  IR: 37 | No significant decrease in PR and MR, but IR decreases with the age | Yes, the impact of age on IR |
|  |  |  |  | 35-42 | PR: 58, MR: 8,  IR: 29 |  |  |
|  |  |  |  | ≥43 | PR: 59, MR: 11,  IR: 30 |  |  |
| Soares et al., 2005 | 3,089 oocyte donation cycles with day 3 ET. | PR, IR | 18-35 | <40 | ^[[3]](#footnote-3)^PR: 48.8, IR: 20.7 | Pregnancy outcome declines after 45 years | Yes, after 45 years of age |
|  |  |  |  | 40-44 | PR: 51, IR: 20.7 |  |  |
|  |  |  |  | 45-49 | PR: 45.5, IR: 17.2 |  |  |
|  |  |  |  | >49 | PR: 35.5, IR: 13.2 |  |  |
| Toner et al., 2002 | Recipients of donated oocytes between 1996 and 1998 (17,339 cycles) | IR, CPR and DR | Not mentioned | 25-29 Fresh ET | CPR: 45.2,  IR: 22.7, DR: 38.9 | Pregnancy outcomes of oocyte donation cycles were not affected by recipients age up to 40 years, after that, there is a decline in fecundity | Yes, after 40 years of age |
|  |  |  |  | 25-29 Cryo-ET | CPR: 32.7,  IR: 13.6, DR: 27.1 |  |  |
|  |  |  |  | 30-34  Fresh ET | CPR: 49.8,  IR: 22.6, DR: 41.8 |  |  |
|  |  |  |  | 30-34 Cryo-ET | CPR: 27.5,  IR: 10.4, DR: 21.8 |  |  |
|  |  |  |  | 35-39 Fresh ET | CPR: 47.2,  IR: 22.6, DR: 40.8 |  |  |
|  |  |  |  | 35-39 Cryo-ET | CPR: 28.1,  IR: 10.4, DR: 21.0 |  |  |
|  |  |  |  | 40-44 Fresh ET | CPR: 48.1,  IR: 22.2, DR: 40.6 |  |  |
|  |  |  |  | 40-44 Cryo-ET | CPR: 28.5,  IR: 10.6, DR: 23.0 |  |  |
|  |  |  |  | 45-49  Fresh ET | CPR: 48.4,  IR: 22.1, DR: 39.6 |  |  |
|  |  |  |  | 45-49 Cryo-ET | CPR: 29, IR: 11.5  DR: 22.2 |  |  |
|  |  |  |  | 50-54  Fresh ET | CPR: 39.7, IR: 15.7, DR: 32.3 |  |  |
|  |  |  |  | 50-54 Cryo-ET | CPR: 24.3, IR: 9.2  DR: 18.6 |  |  |
| Weckstein et al., 1993 | A single-centre study of 69 oocyte donation cycles | Effect of vaginal P4 on CPR in recipients as per age | <35 | < 40 | CPR: 86  IR: 27.7 | The decline in PR was improved after the vaginal P4 | Yes, but it can be reversed |
|  |  |  |  | ≥ 40 | CPR: 24, IR: 9.9  After vaginal P4:  CPR: 66, IR: 32.5 |  |  |
| Yaron et al., 1998 | 1001 oocyte donations and 423 recipients | CPR/ET | <34 | ≤30 | CPR/ET: 36.8 | Age-related decrease in ER. | Yes |
|  |  |  |  | >40 | CPR/ET: 17.8 |  |  |
| Yeh et al., 2014 | Analysis of 27,959 oocyte donation (fresh ET) IVF cycles | CPR, MR, IR and LBR | Mean: 26.3 | ≤34 | ^[[4]](#footnote-4)^CPR: 64.9, IR: 46.5, LBR: 56.7 MR: 16 | Steady and significant decline in PR after the age of 45 years | Yes, after 45 years of age. |
|  |  |  |  | 35-39 | CPR: 65.4, IR: 45.5, LBR: 55.7, MR: 17.7 |  |  |
|  |  |  |  | 40-44 | CPR: 64.7, IR: 45.8, LBR: 55.8, MR: 16.9 |  |  |
|  |  |  |  | 45-49 | CPR: 62.8, IR: 43.4, LBR: 52.7 MR: 18.5 |  |  |
|  |  |  |  | >50 | CPR: 59.9, IR: 40.9, LBR: 48.6, MR: 19 |  |  |

1. # PR after 50mg P4 dose [↑](#footnote-ref-1)
2. φ PR after 100 mg P4 dose [↑](#footnote-ref-2)
3. [↑](#footnote-ref-3)
4. ≥ AR: Abortion rate; CPR: Clinical pregnancy rate; CR: Conception rate; Cu-DR/4ET: Cumulative delivery rate after four embryo transfers; Cu-LBR/4ET: Cumulative live birth rate after four embryo transfers; Cu-PR: Cumulative pregnancy rate; Cu-PR/4ET: Cumulative pregnancy rate after four embryo transfers; DR: Delivery rate; ER: Endometrial receptivity; ET: Embryo transfer; IR: Implantation rate; LBR: Live birth rate; MR: Miscarriage rate; P4: Progesterone; PR: Pregnancy rate [↑](#footnote-ref-4)
