# Supplementary material for: Endometrial receptivity in women of advanced age: an underrated factor in infertility
Source: Hum Reprod Update. 2023 Jul 19;29(6):773–93. doi: 10.1093/humupd/dmad019 (PMC10628506; doi:10.1093/humupd/dmad019)
Supplement: dmad019_Supplementary_Data [file dmad019_supplementary_data.zip › dmad019_Supplementary_Data/Supplementary_table_S2 final.docx]

**Supplementary Table S2** A single pool of oocytes donated by a young woman and distributed among the recipients with young and advanced age (AA).

| **REF** | **Study population** | **Outcome measure** | **Donor age groups/**  **range/**  **Mean age**  **(years)** | **Recipient’s age**  **(years)** | **The outcome in the recipients PR/IR/AR/DR/LBR**  **(%)** | **Conclusion** | **Negative impact on ER**  **Yes/No** |
| --- | --- | --- | --- | --- | --- | --- | --- |
| Navot et al., 1994 | Same-cohort oocytes obtained from one young donor during a specific cycle were evenly distributed between the recipients as per age | CPR, IR, Pregnancy loss. | Mean: 30.2 | <40 | CPR: 21.6,  IR: 13.2,  Pregnancy loss: 9.1 | The conception potential was not correlated with age | No |
|  |  |  |  | ≥40 | CPR: 23.5,  IR: 17.9,  Pregnancy loss: 16.7 |  |  |
| Noyes et al., 2001 | In 122 oocyte donation cycles, donor’s oocytes  were shared between two recipients as per age | CPR and LBR | 18-35 | <35 | CPR: 59, LBR: 45 | No effect of recipient’s age on pregnancy outcome | No |
|  |  |  |  | 35-39 | CPR: 57, LBR: 52 |  |  |
|  |  |  |  | 40-44 | CPR: 60, LBR: 47 |  |  |
|  |  |  |  | ≥45 | PR: 63, LBR: 53 |  |  |
| Cano et al., 1995 | oocytes from the same cohort of follicles were distributed randomly among the recipients as per the age | PR, IR, Pregnancy loss | <35 | <40 | PR: 46.6,  IR: 17.1,  Pregnancy losses: 4.7 | IR was similar in older and younger recipients however; pregnancy outcome was decreased in recipients >40 years | Yes |
|  |  |  |  | ≥40 | PR: 5.5,  IR: 17,  Pregnancy losses: 48 |  |  |
| Harris et al., 2002 | Oocytes were distributed randomly to one, two, or three recipients | PR, LBR | 18-36 | 24–29 | ^[[1]](#footnote-1)^≥PR: 40, LBR: 87 | No effect of recipient’s age on PR, however, the chances of continuing the pregnancy to the full term decreased with age | Yes, the impact of age on LBR but not on PR. |
|  |  |  |  | 30–34 | PR: 45, LBR: 87 |  |  |
|  |  |  |  | 35–39 | PR: 42, LBR: 68 |  |  |
|  |  |  |  | 40–44 | PR: 50, LBR: 69 |  |  |
|  |  |  |  | 45–48 | PR: 45, LBR: 70 |  |  |
|  |  |  |  |  |  |  |  |

1. ≥ AR: Abortion rate; CPR: Clinical pregnancy rate; CR: Conception rate; DR: Delivery rate; ER: Endometrial receptivity; ET: Embryo transfer; IR: Implantation rate; LBR: Live birth rate; MR: Miscarriage rate; P4: Progesterone; PR: Pregnancy rate [↑](#footnote-ref-1)
