# Supplementary material for: Endometrial receptivity in women of advanced age: an underrated factor in infertility
Source: Hum Reprod Update. 2023 Jul 19;29(6):773–93. doi: 10.1093/humupd/dmad019 (PMC10628506; doi:10.1093/humupd/dmad019)
Supplement: dmad019_Supplementary_Data [file dmad019_supplementary_data.zip › dmad019_Supplementary_Data/Supplementary_table_S3 final.docx]

**Supplementary Table S3** Infertile patients undergoing IVF who had shared their oocytes with other recipients of advanced age: Standard IVF with own oocytes vs IVF with donated oocytes.

| **REF** | **Study population** | **Outcome measure** | **Donor age groups/**  **range/**  **Mean age**  **(years)** | **Pregnancy outcome of STD-IVF with own oocytes PR/IR/AR/DR/LBR**  **(%)** | **Recipient’s age groups**  **(years)** | **Pregnancy outcome of DO-IVF with donated oocytes PR/IR/AR/DR/LBR**  **(%)** | **Conclusion** | **Negative impact on ER**  **Yes/No** |
| --- | --- | --- | --- | --- | --- | --- | --- | --- |
| Check et al., 1994 | Shared oocyte donation between 2 groups having an age limit of 40 years | PR and LBR according to age | <40 | CPR: 15.1  LBR: 11.3 | <40 | CPR: 29.6  LBR: 29.6 | The difference in CPR and LBR among younger and older recipients was not statistically significant | No |
|  |  |  | ≥40 | CPR: 14.3  LBR: 7.9 | ≥40 | CPR: 25.4 LBR: 22.4 |  |  |
| Navot et al., 1991 | Donors and recipients undergoing IVF treatment, shared oocytes from the same induced cohort | PR, DR, IR and per embryo transferred | ≤35 | PR: 33  DR: 23  IR: 8.9 | ≥40 | PR: 40  DR: 30  IR: 14.7 | The PR and DR are similar in donors and recipients. | No |
| Borini et al., 1996 | Shared oocyte donation: Total 114 oocyte donors were divided into two groups according to age | PR/ET, IR, AR | <35 | PR: 48.4,  IR: 25.4,  AR: 6.2 | <40 | PR: 47.3  IR: 24.8  AR: 14.8 | The advanced age has an impact on PR and IR | Yes |
|  |  |  |  |  | 40-49 | PR: 24.5  IR: 14.9  AR: 7 |  |  |
| Braga et al., 2020 | Oocyte sharing program: 1,505 vitrified oocytes donated from 268 patients to 225 oocyte recipients, undergoing 307 ICSI cycles | PR and IR. | Range: 19-34 | PR: 47.1  IR: 29.4±40.1 (mean ± SD) | Range: 26-50 | PR: 39.5  IR: 29.6 ± 40.4 (mean ± SD) | The age of oocyte recipients was negatively correlated with pregnancy outcome | Yes |
| Check et al., 1993 | Oocytes from infertile women undergoing IVF-ET were shared with younger and older recipients with age limit of 40 | PR | <40 | Not mentioned | <40 | PR: 25.4 | The low pregnancy rate in the older patients suggests the decreased uterine receptivity | Yes |
|  |  |  | ≥40 | Not mentioned | ≥40 | PR: 8.5 |  |  |
|  |  |  | ≥40 | CPR: 12.9, MR: 30.9 | ≥40 | CPR: 21.2, MR: 18 |  |  |
| Levran et al., 1991 | 91 oocyte donation cycles and 169 recipients. Oocytes were shared between STD-IVF and DO-IVF | CR, MR | ≤31 | PR: 78  MR: 22 | ≤32 | CR: 31.4 | Endometrial aging is responsible for reduced fecundity | Yes |
|  |  |  | ≥32 | PR: 30  MR: 70 | ≥33 | CR: 12.6 |  |  |

AR: Abortion rate; CPR: Clinical pregnancy rate; CR: Conception rate; DO-IVF: Donor IVF; DR: Delivery rate; ER: Endometrial receptivity; ET: Embryo transfer; IR: Implantation rate; LBR: Live birth rate; MR: Miscarriage rate; P4: Progesterone; PR: Pregnancy rate; PR/ET: pregnancy rate per embryo transfer; SD: standard deviation; STD-IVF: Standard IVF with own oocytes
